# Expression of acyl-CoA-binding protein 5 from *Rhodnius prolixus* and its inhibition by RNA interference

**DOI:** 10.1371/journal.pone.0227685

**Published:** 2020-01-14

**Authors:** Muriel G. M. D. Almeida, Daniela S. Arêdes, David Majerowicz, Nils J. Færgeman, Jens Knudsen, Katia C. Gondim

**Affiliations:** 1 Instituto de Bioquímica Médica Leopoldo de Meis, Universidade Federal do Rio de Janeiro, Rio de Janeiro, Brazil; 2 Institut for Biokemi og Molekylær Biologi, Syddansk Universitet, Odense, Denmark; 3 Faculdade de Farmácia, Universidade Federal do Rio de Janeiro, Rio de Janeiro, Brazil; Instituto Oswaldo Cruz, BRAZIL

## Abstract

The acyl-CoA-binding proteins (ACBP) act by regulating the availability of acyl-CoA in the cytoplasm and must have essential functions in lipid metabolism. The genome of the kissing-bug *Rhodnius prolixus* encodes five proteins of this family, but little is known about them. In this study we investigated the expression and function of RpACBP-5. Feeding induced *RpACBP-5* gene expression in the posterior midgut, and an increase of about four times was observed two days after the blood meal. However, the amount of protein, which was only detected in this organ, did not change during digestion. The *RpACBP-5* gene was also highly expressed in pre-vitellogenic and vitellogenic oocytes. Recombinant RpACBP-5 was shown to bind to acyl-CoA of different lengths, and it exhibited nanomolar affinity to lauroyl-CoA in an isothermal titration assay, indicating that RpACBP-5 is a functional ACBP. *RpACBP-5* knockdown by RNA interference did not affect digestion, egg laying and hatching, survival, or accumulation of triacylglycerol in the fat body and oocytes. Similarly, double knockdown of RpACBP-1 and RpACBP-5 did not alter egg laying and hatching, survival, accumulation of triacylglycerol in the fat body and oocytes, or the neutral lipid composition of the posterior midgut or hemolymph. These results show that RpACBP-5 is a functional ACBP but indicate that the lack of a detectable phenotype in the knockdown insects may be a consequence of functional overlap of the proteins of the ACBP family found in the insect.

## Introduction

Acyl-CoA binding proteins (ACBP) are considered the central protein family that binds esterified fatty acids (FA), acting in the subtle control of their intracellular concentration. These proteins form a gene family containing proteins of different sizes which present an acyl-CoA-binding domain [1]. ACBPs are highly conserved in all species of eukaryotes and prokaryotes hitherto analyzed, and they are predominantly cytosolic proteins that bind acyl-CoAs in a non-covalent and reversible way. They have high affinity and specificity for medium- and long-chain saturated or unsaturated acyl-CoAs, with *K*_*d*_ varying from 1 to 15 nM [2]. ACBPs are generally expressed in all tissues of an organism, which, considering the high degree of conservation between species, points to the idea that this protein is involved in processes that are necessary for the maintenance of primary cellular function [3]. However, the precise biological functions that this family of proteins exerts are just beginning to be unraveled through gene silencing or inactivation assays.

ACBP knockdown by small interference RNA caused a significant decrease in FA levels in human hepatocytes [4]. The synthesis of sphingolipids and ceramides also appears to be regulated by these proteins, since the *Acb1* deletion depleted these compounds in the yeast *Saccharomyces cerevisiae* [5]. Moreover, the deletion of either the *Acb1* gene of the yeast or the membrane-associated ACBP gene of the nematode *Caenorhabditis elegans* resulted in the disruption of the cell membrane morphology, and generated cells with multilobed vacuoles, invaginations, and accumulation of vesicles of various sizes. Autophagocytic corpuscles, membrane fragments, and membrane structures with more than two phospholipid layers were also observed. These results indicate that ACBP modulates vesicle traffic, organelle biogenesis and membrane assembly [5,6]. The *Acbp2* gene deletion in *C*. *elegans* caused a dramatic decrease in the degradation of unsaturated FAs via the β-oxidation route [7], indicating the importance of ACBP in lipid degradation. Regarding the regulation of gene expression, ACBP modulates the expression and activation of specific genes and transcription factors, such as HNF-4α, PPARγ, and SREBP-1, causing changes in the expression profile of lipid metabolism genes [4,5,8–10]. ACBP also participates in apoptosis in rodents [11], and is associated with the maintenance of the epidermal barrier of mice [12]. Finally, in mice ACBP seems to be essential, as its deletion is lethal, reinforcing the idea that these proteins play a fundamental role in cell metabolism [13].

However, there is little information on the role of ACPBs in insects. In the silkworm *Bombyx mori*, ACBP knockdown in the pheromone gland reduced both the amount of triacylglycerol (TAG) stored in the gland, and the pheromone bombykol synthesis [14]. Also, the gene *Anorexia* deletion in the fruit fly *Drosophila melanogaster* showed that this gene is necessary for gustatory sensation and control of food intake, through the regulation of insulin signaling by a possible modulation of the insulin receptor expression in the nervous system [15].

The kissing-bug *Rhodnius prolixus* is a hematophagous hemipteran and one of the primary vectors of Chagas' disease, which infects about 8 million people in Central and South America [16]. In addition to its medical importance, this insect is widely used as a model for studies of biochemistry and physiology, including lipid metabolism [17]. Analysis of the *R*. *prolixus* genome revealed that it encodes five different ACBPs [18]. Previous studies showed that *RpACBP-1* expression in the posterior midgut increases about 10-fold on the first day after feeding. The release of serotonin in the hemolymph modulates this induction, through a signaling pathway involving stimulatory G protein and cyclic AMP [19]. Also, a native gel migration assay revealed that recombinant RpACBP-1 bound acyl-CoA of different carbon lengths *in vitro*. This gene knockdown by RNA interference (RNAi) caused an accumulation of TAG and a concomitant decrease of diacylglycerol (DAG) content in the insect posterior midgut. On the other hand, the amount of TAG stored in the flight muscle was reduced. In the fat body, the incorporation of FA from the hemolymph into cholesterol esters (CE) was increased [18]. About the other four ACBP genes, the only available information concerns their expression profiles. The *RpACBP-2* gene is mostly expressed in the flight muscle, whereas the *RpACBP-5* gene is highly expressed in the posterior midgut and ovaries. All analyzed organs expressed *RpACBP-3* and *4* at similar levels [18]. It is important to note that *RpACBP-1* and *5* are around 10 kDa and only have the ACBP domain in their primary sequences, while the other proteins are larger and have other associated domains [18].

Herein, the gene and protein expression profiles and the binding capacity of RpACBP-5, whose amino acid sequence is similar to that of RpACBP-1, were characterized, and its possible roles in the metabolism of *R*. *prolixus* were also investigated.

## Material and methods

### Insects

The insects used in this study were adult mated females or males (only for testis), on the third feeding cycle, from a colony maintained at 28°C and 70–75% relative humidity. Adults were fed at 21-day intervals on live rabbit blood. Animal care followed protocol number 155/13 approved by the local committee on ethics in animal experimentation, Comissão de Ética no Uso de Animais–Universidade Federal do Rio de Janeiro (CEUA-UFRJ), and the recommendations of the NIH Guide for the Care and Use of Laboratory Animals (ISBN 0-309-05377-3).

### Quantitative PCR (qPCR)

At appropriate days after feeding, the organs of adult females were dissected and washed in PBS (10 mM sodium phosphate, pH 7.4, 150 mM NaCl), and immediately homogenized (pools of three organs) in TRIzol Reagent (Invitrogen, Carlsbad, CA, USA). Total RNA was extracted according to the manufacturer's protocol and quantified with NanoDrop Lite Spectrophotometer (Thermo Fisher Scientific, Waltham, MA, USA). The integrity and quality of the RNA samples were analyzed by electrophoresis on 1% agarose gel (UBS, Cleveland, OH, USA). RNA was considered intact when the 18S rRNA band was observed. All samples showed the A260/A280 ratio between 1.9 and 2.0. After extraction, 1 μg of total RNA was treated with 1 U of DNAse I (Sigma-Aldrich, Saint Louis, MO, USA) for 30 min at 37°C in a final volume of 10 μl. The reaction was stopped with incubation at 70°C for 10 min and addition of 50 mmol of EDTA. All treated RNA was used as template for cDNA synthesis with random primers and 50 U of MultiScribe^™^ MuLV reverse transcriptase (High Capacity cDNA synthesis kit; Thermo Fisher Scientific), in a final volume of 22 μl, at 37°C for 2 h. To confirm DNAseI treatment efficiency, control reactions were done without addition of reverse transcriptase. The qPCR was done in a StepOnePlus thermocycler (Thermo Fisher Scientific), using GoTaq qPCR Master Mix (Promega, Madison, WI, USA). The reaction mixture contained three pmol of sense and antisense primers in a final volume of 15 μl, as follows: 10 min at 95°C, followed by 40 cycles of 15 s at 95°C and 1 min at 60°C, and a dissociation curve. Primer sequences and additional information are provided in S1 Table and S2 Table. For the blanks, the cDNA was replaced by nuclease-free water. The Cq values obtained for the blanks were at least ten units above the experimental points. The Cq values obtained for the controls (cDNA synthesis reaction without reverse transcriptase) were at most five units below the blanks. The amplification of the *Rp18S* or *RpEF-1* genes were used as reference genes, as previously described [20], and it was confirmed that their expression was constant under our experimental condition [21]. The ΔΔCq values were calculated by the Cq values obtained as described in the literature [22]. These values were used for statistical analyses. The relative expression values (2^-ΔΔCq^) were used only for data plotting.

### Heterologous expression

The *RpACBP-5* gene (Genbank accession number KC417418; sequence length 261 bp) was heterologously expressed in bacteria as previously described for *RpACBP-1* (Genbank accession number EU233793; sequence length 267 bp) [18,23]. Briefly, the *RpACBP-5* gene was subcloned into the expression vector pACYCDuet-1 (Merck KGaA, Darmstadt, Germany), together with the *methionine aminopeptidase* gene from *Escherichia coli*. The resulting expression vector was used to transform *E*. *coli* BL21 (DE3). The transformed bacteria was grown in LB medium (1% w/v peptone, 0.5% w/v yeast extract, 1% w/v NaCl) at 37°C and 200 rpm until reaching O.D. 1.0, when the heterologous protein expression was induced by the addition of 100 mM IPTG (isopropyl β-d-1-thiogalactopyranoside) for 3 h. Cells were collected and lysed with an Ultra Turrax T25 homogenizer (IKA-Works, Inc. Wilmington, NC, USA) and sonication. Proteins were precipitated with 50% trichloroacetic acid, and the recombinant protein was purified by gel filtration chromatography, using a G50 column (GE Healthcare, Little Chalfont, UK), followed by ion exchange chromatography, using a Q Sepharose column (GE Healthcare).

### Immune serum production

A rabbit was injected with 1 mg of recombinant RpACBP-5 containing 25% complete Freund's adjuvant and 25% incomplete Freund's adjuvant. After 30 days, the immunization was boosted with the injection of 0.5 mg of the recombinant protein under the same conditions. Fifteen days later, the immune serum was obtained, aliquoted and stored at -20°C.

### Immune serum cross-reaction test

Different amounts of recombinant RpACBP-5 were applied onto a nitrocellulose membrane (GE Healthcare), along with 10 μg of recombinant RpACBP-1 [18], to determine antibody specificity. The membrane was blocked for 1 h with TBSTM buffer (10 mM Tris-HCl, pH 8.0, 150 mM NaCl, 0.1% Tween 20, 5% skim milk) at room temperature, and incubated with the anti-RpACBP-5 serum at 1:300 dilution in TBSTM. The membrane was then washed with TBST buffer (10 mM Tris-HCl, pH 8.0, 150 mM NaCl, 0.1% Tween 20) and incubated with peroxidase-coupled anti-rabbit IgG antibody (Santa Cruz Biotechnology, Santa Cruz, CA, USA), at 1:1000 dilution in TBSTM. The membrane was again washed with TBST buffer and developed with Luminata^™^ Strong Western HRP Substrate (Millipore, Billerica, MA, USA). The membrane was then scanned with C-DiGit^®^ Blot Scanner (LI-COR Biosciences, Lincoln, NE, USA).

### Western blot

The insects were dissected and the obtained organs were homogenized in homogenization buffer (5 mM Tris-HCl, pH 7.4) containing 2 mM PMSF (phenylmethylsulfonyl fluoride), 5 mM NaF, 2 mM VO_4_^-3^, and 1% protease inhibitor cocktail (Sigma-Aldrich). Hemolymph was collected into a tube containing a few grains of phenylthiourea, by cutting the first pair of legs. After homogenization, samples were centrifuged at 14,000 *g* at room temperature for 5 min. Supernatants were collected, and total protein concentration was determined [24]. Samples (60 μg protein) were loaded onto a 10% polyacrylamide gel, and recombinant RpACBP-5 was used as a positive control. The electrophoresis was run with cathode buffer (100 mM Tris, pH ~ 8.25, 100 mM tricine, 0.1% SDS) and anode buffer (100 mM Tris-HCl, pH 8.9) at 125 V for 45 min. Proteins were transferred to a nitrocellulose membrane using transfer buffer (25 mM Tris-HCl, pH 8.3, 192 mM glycine, 20% methanol) at 45 V for 90 min. The membrane was blocked and incubated with primary antiserum and secondary antibody as described above. The primary monoclonal anti-α-tubulin antibody (Santa Cruz Biotechnology) at 1:1000 dilution, followed by peroxidase-coupled anti-mouse IgG antibody (Santa Cruz Biotechnology), was used as loading control. The intensity of the bands was analyzed by densitometry with Image J software version 1.50i (NIH Image, Bethesda, MD, USA), with background corrections.

### Analysis of acyl-CoA binding by native gel assay

The binding of acyl-CoA to RpACBP-5 was analyzed *in vitro* by native polyacrylamide gel as previously described [18,25]. Briefly, one nmol of the recombinant protein was mixed with 8 nmol of the acyl-CoA in sample buffer (12 mM sodium phosphate, pH 7.4, 82.2 mM NaCl, 0.1% bromophenol blue, 5% glycerol). Acyl-CoAs were purchased from Avanti Polar Lipids, Inc. (Alabaster, AL, USA). Samples were separated on native 8–16% polyacrylamide gel (Invitrogen), in running buffer (25 mM Tris, pH 8.3, 192 mM glycine) at 125 V for 100 min. The gel was stained with Coomassie blue.

### Isothermal titration calorimetry

The binding assay was performed in the microcalorimeter as previously described [26]. Briefly, lipid binding to the recombinant RpACBP-5 was analyzed in a VP-ITC microcalorimeter (MicroCal, Boston, MA, USA) equilibrated at 27°C. The protein solution (25 μM in 25 mM ammonium acetate buffer, pH 6.0) was loaded into the calorimeter cell and titrated with 0.5 M lauroyl-CoA in the same buffer, using 30 consecutive aliquots of 4 μl at 3 min interval. The solution was mixed at 400 rpm. The obtained data were analyzed by Origin software (MicroCal).

### RNA interference

The double strand RNAs (dsRNAs) for the complete sequences of the *RpACBP-1* and *RpACBP-5* genes (S3 Table) were produced with the MEGAScript^™^ T7 High Yield Transcription Kit (Thermo Scientific), according to the manufacturer's protocol. dsRNA for an unrelated control gene was also produced. A fragment of 808 bp of the *E*. *coli MalE* gene (Gene ID: 948538), included in the control plasmid LITMUS 28iMal obtained from the HiScribe RNAi Transcription kit (New England BioLabs), was amplified by PCR using a T7 promoter-specific primer targeting the opposing T7 promoters of the vector. The cycling conditions were: 5 min at 94°C, followed by 40 cycles of 30 s at 94°C, 30 s at 44°C and 1 min at 72°C, and a final extension of 10 min at 72°C. The amplified fragment was used as a template for the control dsRNA (dsMal). Different amounts of dsRNA were injected into the insect hemocoel using a 10 μl microsyringe (Hamilton Company, Reno, NV, USA). Insects were dissected at different days after injection, and inhibition of expression was confirmed by qPCR as described above.

### Measurement of digestion

The insects had the midgut (anterior plus posterior midguts) dissected on days 1, 2, 4, 7 and 10 after feeding, to follow the protein digestion. Immediately after dissection, the organs were exposed to liquid nitrogen and stored at -70°C until analysis, when they were individually homogenized in PBS containing 1% protease inhibitor cocktail (Sigma-Aldrich), for the determination of total protein content [24].

### Enzymatic quantification of TAG

Insects were dissected on the seventh day after feeding, and the fat body and chorionated oocytes were obtained and homogeneized in PBS buffer. For TAG content determination, the Triglicérides 120 colorimetric Kit (Doles Reagents, Goiânia, Brazil) was used. Total protein content was also determined (Lowry et al., 1951).

### Oviposition, hatching, and survival

For oviposition analysis, females were separated in individual vials immediately after feeding. The laid eggs were daily collected and counted until the end of the laying cycle. Hatching was also accompanied, and nymph morphology was visually inspected. For comparison of the adult lifespan, these females were daily observed until all the insects had died.

### Lipid composition analysis

Hemolymph was collected in the fourth day after feeding, as described in section “Western blot”, to determine the lipid composition. In addition to hemolymph, the posterior midgut was also dissected, cleaned in cold PBS buffer for removal of the luminal contents, and homogenized in PBS buffer containing 2 mM PMSF and 1% protease inhibitor cocktail (Sigma-Aldrich). The samples were centrifuged at 3,000 *g* at room temperature for 5 min. The supernatant was collected, and protein concentration was determined as described. The samples were subjected to lipid extraction in chloroform as described [27]. Neutral lipid composition was analyzed by high-performance thin-layer chromatography (HPTLC) on silica gel plates (Merck), using two consecutive solvent systems, as described elsewhere [28]. The lipid relative composition was determined by densitometry with the Image J program (NIH Image) with background corrections, and their identification was performed by comparison with commercial lipid standards (Sigma Aldrich).

## Results

**Fig 1:**
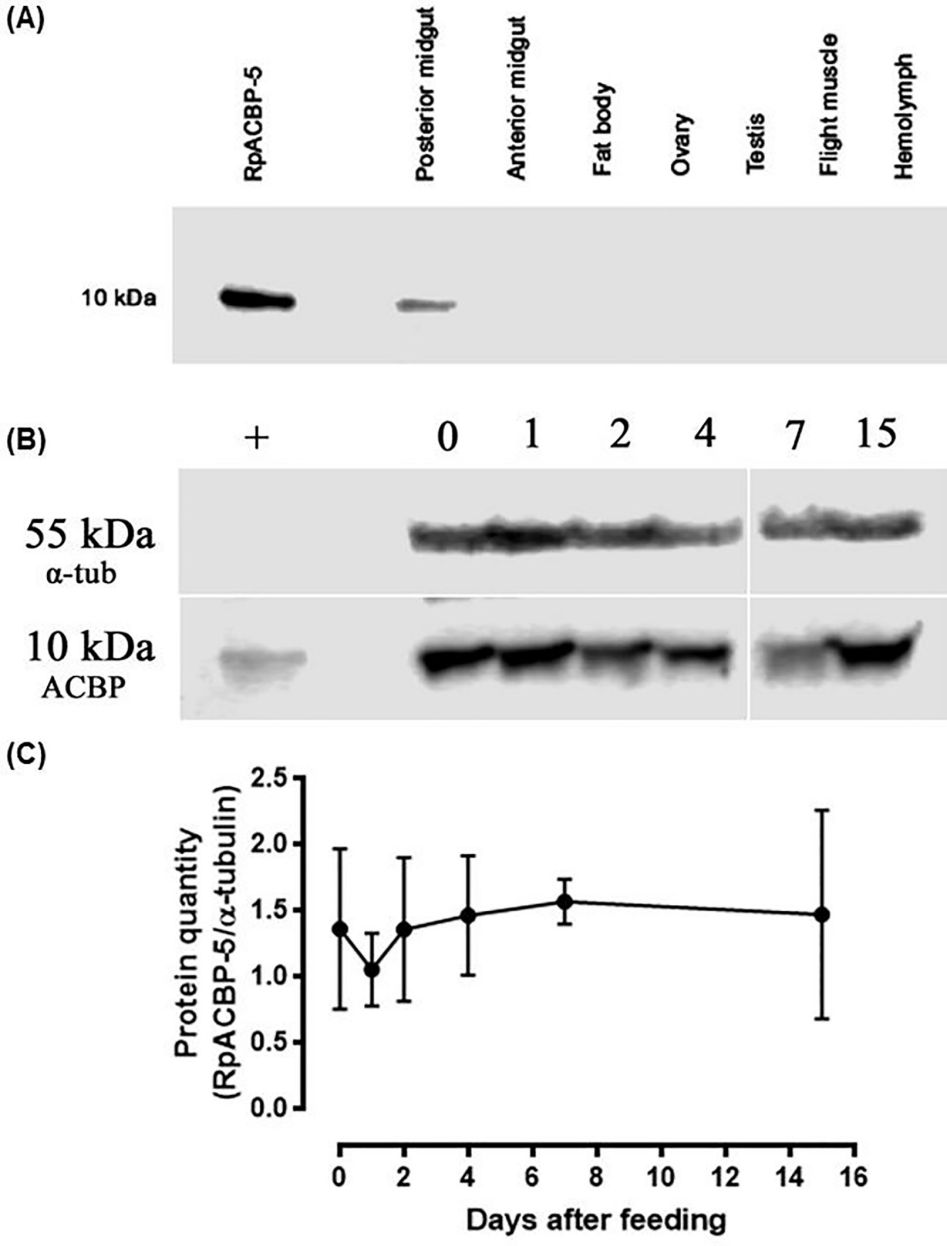
The RpACBP-5 protein shows stable expression level in the posterior midgut after feeding. (A) Females and males (testis) were dissected on the 4^th^ day after feeding, and the midgut, hemolymph, fat body, ovary, testis and flight muscle were obtained. (B) Females were dissected before feeding (day 0) or at 1, 2, 4, 7, and 15 days after blood meal, and the posterior midgut was obtained. After homogenization, samples (60 μg of protein) were subjected to Western blotting, and the anti-RpACBP-5 antiserum or anti-αtubulin antibody were used. Recombinant RpACBP-5 (20 ng) was used as a positive control. The images are representative of three experiments. (C) The intensity of the bands was determined by densitometry. The symbols are means ± SEM. *p* > 0.05 by one-way ANOVA, n = 3.

In a previous study, where RpACBP genes were analyzed, it was shown that *RpACBP-5* was predominantly expressed in the posterior midgut and ovary [18]. So, to evaluate whether protein expression followed this same pattern, the presence of RpACBP-5 in the posterior and anterior midgut, fat body, ovary, testis, flight muscle and hemolymph of females and males (testis) in the fourth day after feeding was examined by Western blot. It was detected only in the posterior midgut (Fig 1A), where it was present in all analyzed conditions, from fasting to the 15^th^ day after blood meal (Fig 1B). It could also be observed that the protein amount remained constant, without any significant variations throughout the digestion period (Fig 1C). It is important to note that the used antiserum was specific for RpACBP-5, since it detected 100 ng of the recombinant protein in a dot blot assay, but not the recombinant RpACBP-1, even when 100 times more protein was used (S1 Fig).

**Fig 2:**
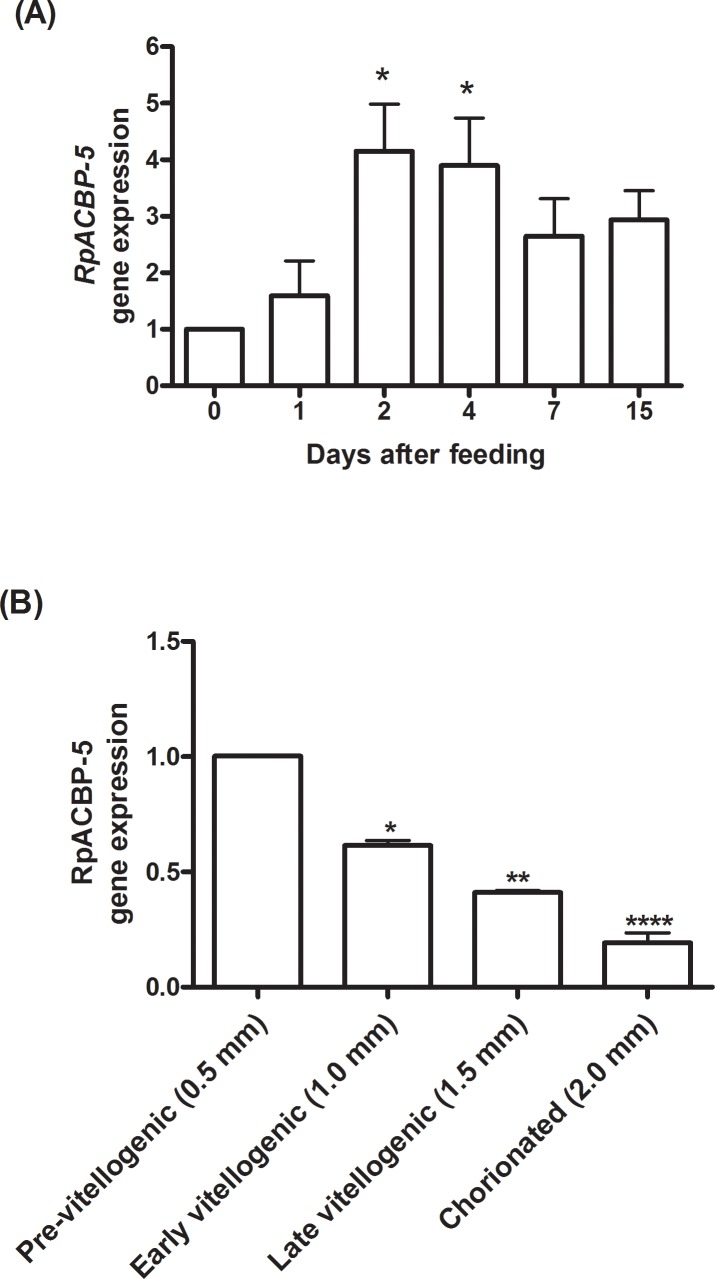
The *RpACBP-5* gene is induced after feeding in the posterior midgut and it is highly expressed in the pre-vitellogenic oocytes. (A) The posterior midgut was obtained from fasted *R*. *prolixus* females (day 0) or on days 1, 2, 4, 7, and 15 after feeding. The *RpACBP-5* mRNA levels were quantified by qPCR, using the *RpEF-1* expression as reference gene. The bars are means ± SEM. (*) *p* < 0.05 when compared to fasted insects by one-way ANOVA followed by Tukey's post-test, n = 4 (except day 1, n = 3). (B) Females were dissected on the fourth day after feeding, the ovary was removed and the follicles separated by size (length).The *RpACBP-5* mRNA levels were quantified by qPCR, using the *Rp18S* expression as reference gene. The bars are means ± SEM. (*), (**), (****) significantly different from pre-vitellogenic follicles by one-way ANOVA, followed by Tukey's post-test with *p* < 0.05, 0.01, and 0.0001, respectively; n = 3 (except 0.5 mm, n = 4).

However, although RpACBP-5 protein amount in the posterior midgut was stable, *RpACBP-5* mRNA level increased after blood meal, and was maximal between the second and fourth days, when it reached levels about four times higher than in the fasted insect (Fig 2A). At the seventh day, the expression decreased, and was at the same level at the 15^th^ day after feeding. Because *RpACBP-5* expression was also high in the ovary [18], transcript levels were evaluated during oocyte development. The mRNA amount was maximal in the 0.5 mm follicles, and decreased as oocytes evolved. Transcript levels were reduced to 40% and 20% of the initial values in the 1.5 and 2.0 mm follicles, respectively (Fig 2B).

**Fig 3:**
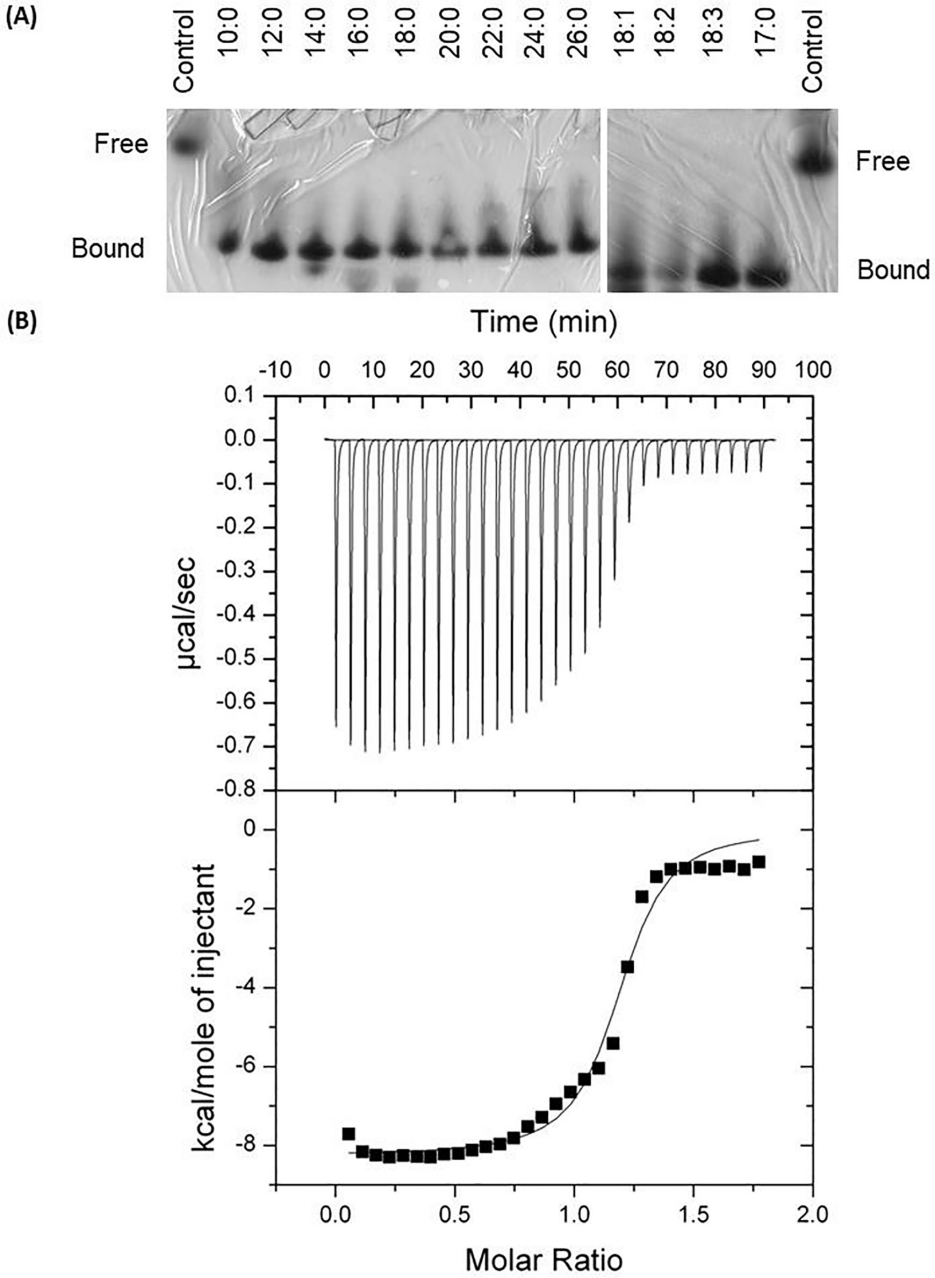
RpACBP-5 binds acyl-CoA *in vitro*. (A) The purified recombinant RpACBP-5 was added to the acyl-CoA and binding buffer and then subjected to a native polyacrylamide gel electrophoresis. Controls did not receive acyl-CoA. Numbers indicate the size of the fatty acid carbon chain followed by the number of unsaturations, present on the tested acyl-CoA. The images are representative of two experiments. (B) Lauroyl-CoA binding to RpACBP-5 was analyzed in microcalorimeter equilibrated at 27°C. Protein (25 μM) was placed in the calorimeter chamber and titrated with 0.5 mM lauroyl-CoA, in binding buffer. There were 30 injections of 4 μl every 180 seconds. A representative result of five experiments is shown.

The RpACBP-5 capacity to bind acyl-CoA of different chain lenghts was first evaluated by a native gel migration assay. As shown in Fig 3A, RpACBP-5 bound acyl-CoA ranging in size from 10 to 26 carbons, as well as acyl-CoA with up to three unsaturations, or with an odd number of carbons. The acyl-CoA binding to RpACBP-5 was then investigated by isothermal titration calorimetry, using lauroyl-CoA as titrant. Lauroyl-CoA bound to RpACBP-5 with a significant heat release (about -0.65 μcal/sec) and, when the acyl-CoA/ACBP molar ratio reached 1, the protein rapidly saturated and binding heat was lower than -0.1 μcal/sec (Fig 3B). The binding stoichiometry (n) was 1.190 ± 0.05, indicating that each RpACBP-5 molecule binds one acyl-CoA. The measured dissociation constant (*K*_*d*_) was 399 ± 266 nM, showing that RpACBP-5 has an affinity to acyl-CoA at the nanomolar range. The calculated change in enthalpy (ΔH) was -8086.8 ± 417.28 J/mol and the change in entropy (ΔS) was 2.778 ± 1.27 J/K. Thus, it can be concluded that RpACBP-5 functions as an acyl-CoA binding site in the posterior midgut, interacting with the lipid even at low intracellular concentrations.

**Fig 4:**
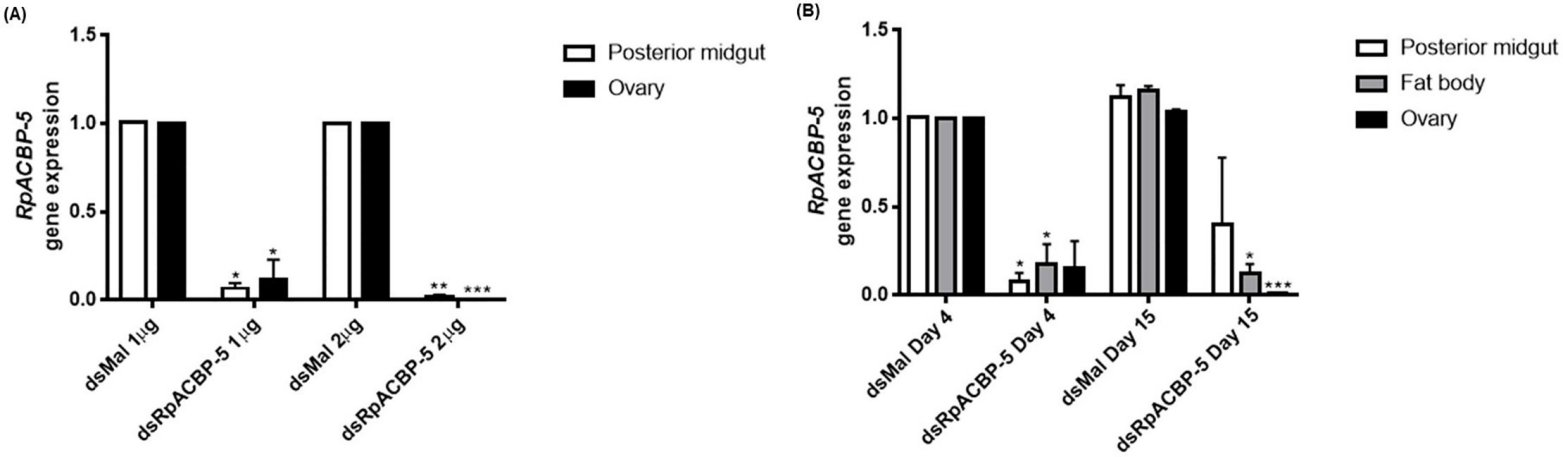
*RpACBP-5* knockdown by dsRNA injection. Fasting adult females were injected with 1 or 2 μg of dsRNA against *RpACBP-5* (dsRpACBP-5) or *Mal* gene (dsMal), and dissected on the third day after injection. Total RNA was extracted from the posterior midgut and ovary (A). Alternatively, fasting females were injected with 1 μg of dsRpACBP-5 or dsMal, fed three days later, and dissected at the fourth and 15^th^ day after feeding (B). Total RNA was extracted from the posterior midgut, fat body, and ovary. The *RpACBP-5* mRNA levels were quantified by qPCR, using the *Rp18S* expression as reference gene. The bars are means ± SEM. (*), (**) and (***): significantly different from dsMal by Student's *t*-test with *p* < 0.05, 0.01, and 0.001, respectively. (A) n = 5 or n = 3, for 1 and 2 μg, respectively. (B) n = 3.

**Fig 5:**
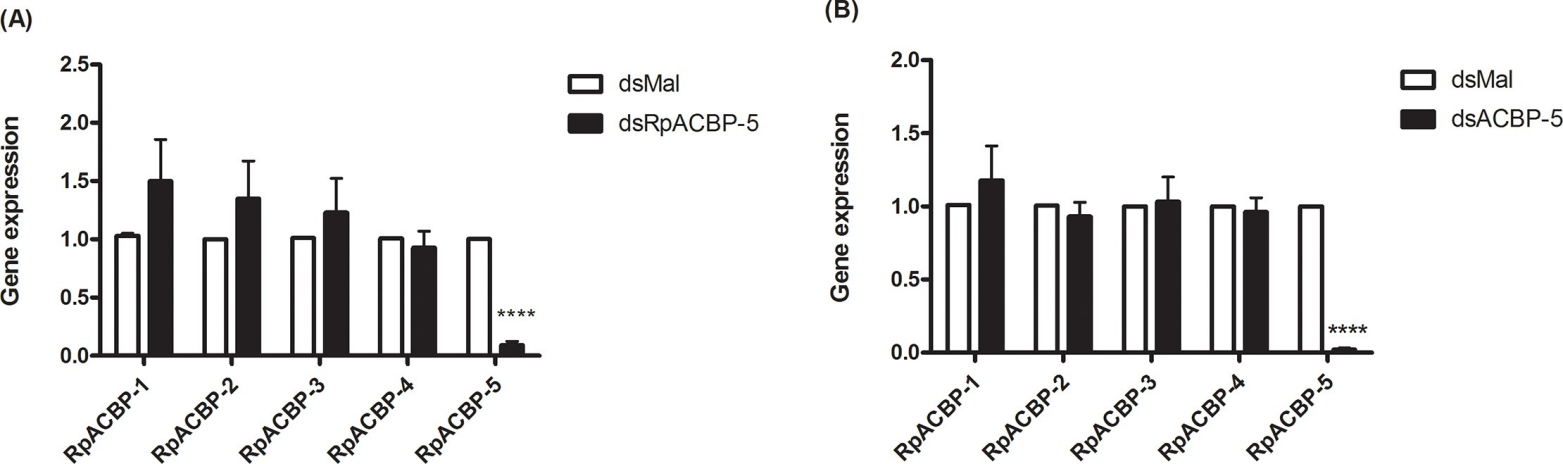
*RpACBP-5* knockdown does not significantly affect expression of the other *RpACBP* genes. Fasting adult females were injected with 1 μg of dsRpACBP-5 or dsMal, fed three days later and dissected at the fourth day after blood meal. Posterior midguts (A) and ovaries (B) were obtained and homogenized. Samples were subjected to qPCR, for determination of *RpACBP-1*, *RpACBP-2*, *RpACBP-3*, *RpACBP-4*, and *RpACBP-5* mRNA levels, using *Rp18S* expression as reference gene. The bars are means ± SEM. (****): significantly different from dsMal ​​by Student's *t*-test with *p* < 0.0001; n = 8 (A) and n = 7 (B).

RpACBP-5 expression was inhibited by RNAi. The treatment with 1 μg of dsRNA caused an expression reduction of 93% in the posterior midgut and 85% in the ovary, three days after injection, in unfed females. With 2 μg, both organs showed inhibition above 98% (Fig 4A). As inhibition was very effective with 1 μg injection, we proceeded with this amount. This decrease in gene expression was maintained after the blood meal, and relevant decreases in the amount of mRNA were confirmed up to 15 days after feeding, in posterior midgut, ovary, and fat body. In posterior midgut, a sixfold decrease in transcripts was observed when compared to the control, on the 4^th^ day. In the ovary, a 99% gene expression inhibition was observed 15 days after feeding (Fig 4B). It was possible that *RpACBP-5* knockdown affected the expression of other ACBP genes, but no increase in any *RpACBP* gene expression was detected under the analyzed conditions. The variations observed in some transcript levels were not statistically significant, indicating that the obtained effect of RNAi was specific for *RpACBP-5* (Fig 5).

**Fig 6:**
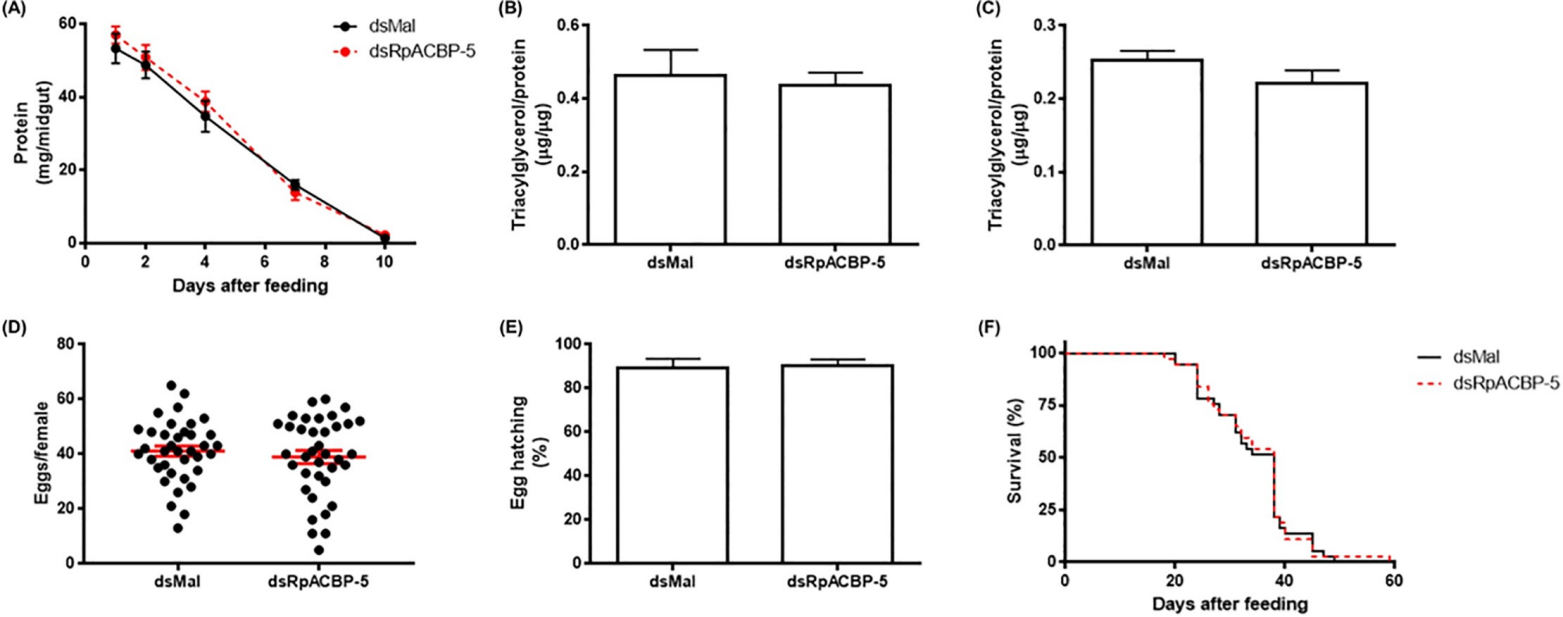
*RpACBP-5* knockdown does not affect digestion, lipid accumulation, reproduction or lifespan. Adult fasted females were injected with one μg of dsRpACBP-5 or dsMal, and insects were fed three days post-injection. The symbols and bars are means ± SEM. (A) Insects were dissected on different days after feeding, and the total protein amount in the midgut was determined. *p* > 0.05 by two-way ANOVA, n = 9. The fat body (B) and oocytes (C) were collected on the seventh day after feeding, and the TAG content in samples was determined. *p* > 0.05 by Student's *t*-test, n = 8. (D) Oviposition was individually monitored throughout the days after feeding. *p* > 0.05 by Student's *t*-test, n = 37. (E) Eggs were collected on different days of the oviposition cycle. The total number of hatched eggs in each experimental group was determined. *p* = 0.0776, by χ^2^ test, n = 1475. (F) After blood meal, insects were monitored daily. *p* > 0.05 by Log-Rank test, n = 37.

Since, among all ACBP genes, *RpACBP-5* is the most abundantly expressed in the posterior midgut [18], it was possible that *RpACBP-5* knockdown affected the insect digestion. However, no difference was observed in knockdown insects, when digestion rate was analyzed (Fig 6A). *RpACBP-5* knockdown could also interfere with the absorption and transport of lipids from the midgut to the organs that use them and, in this way, affect the accumulation of lipids by the fat body, as well as the production and viability of eggs. In order to check this possibility, the TAG amount in the fat body and oocytes was quantified, and no difference was observed (Fig 6B and 6C). Moreover, to verify any possible impact that the decrease in RpACBP-5 levels could have in reproduction, the number of laid eggs and the rate of hatching were determined. Although the number of eggs laid by the knockdown animals was about 8% lower, this difference is too small and is not significant (Fig 6D). Both groups showed hatching percentages above 89% (Fig 6E). In this way, *RpACBP-5* knockdown did not interfere with oogenesis or nymph development during embryogenesis. The insect longevity was then monitored in starvation condition, and the gene knockdown had no detectable effect (Fig 6F).

**Fig 7:**
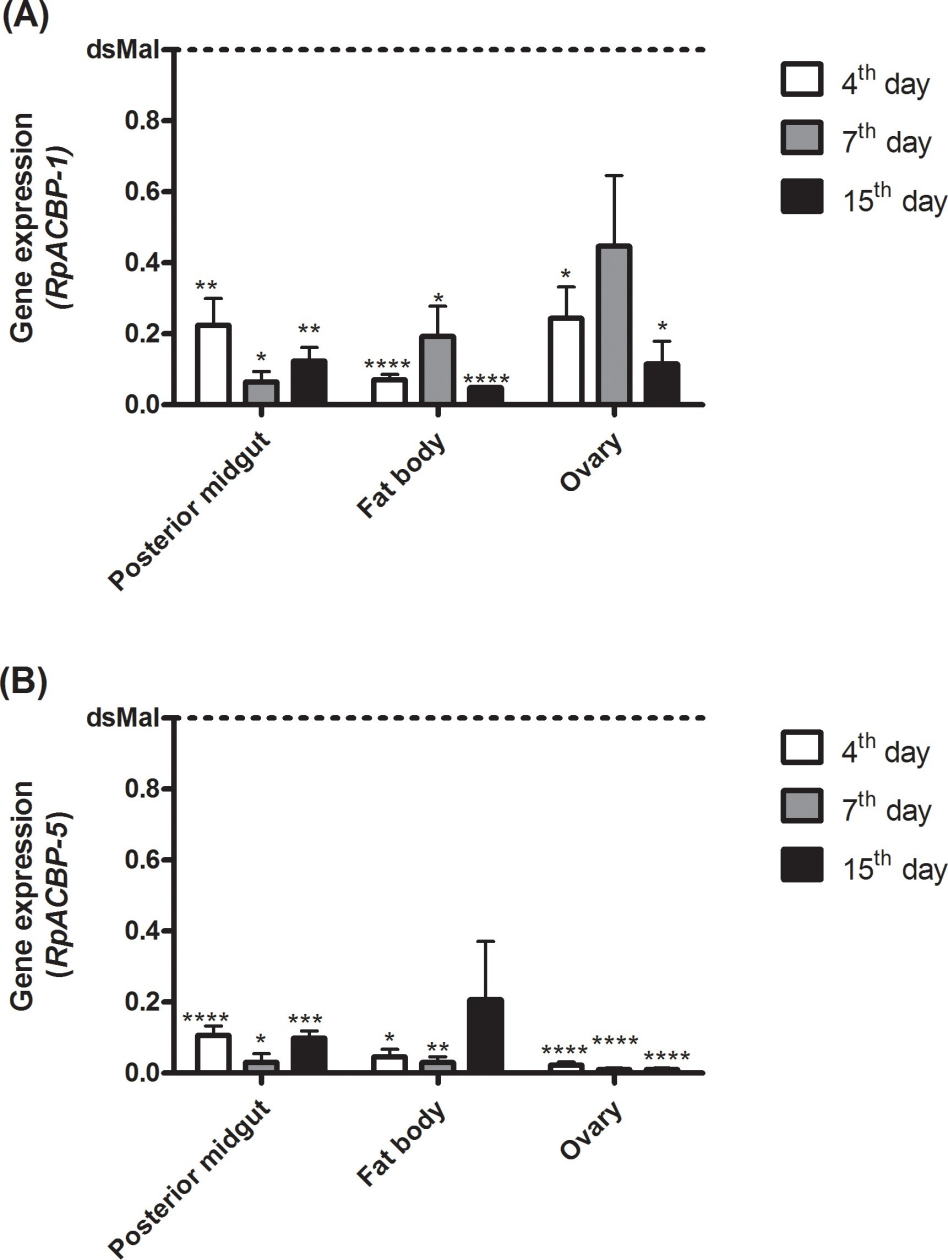
*RpACBP-1* and *RpACBP-5* gene expression after double knockdown by RNAi. Fasting adult females were injected with a mixture of dsRNAs containing 1 μg of dsRpACBP-1 and 1 μg of dsRpACBP-5 or with 2 μg of dsMal, used as a control. The insects were fed three days after injection and dissected on the 4^th^, 7^th^ and 15^th^ day after feeding. The *RpACBP-1* and *RpACBP-5* mRNA levels were quantified by qPCR, using the *Rp18S* expression as a reference gene. The bars are means ± SEM. (*), (**), (***), and (****): significantly different from dsMal by Student's *t*-test with *p* < 0.05, 0.01, 0.001, and 0.0001, respectively; n = 3–4. (A) *RpACBP-1* gene expression. (B) *RpACBP-5* gene expression.

**Fig 8:**
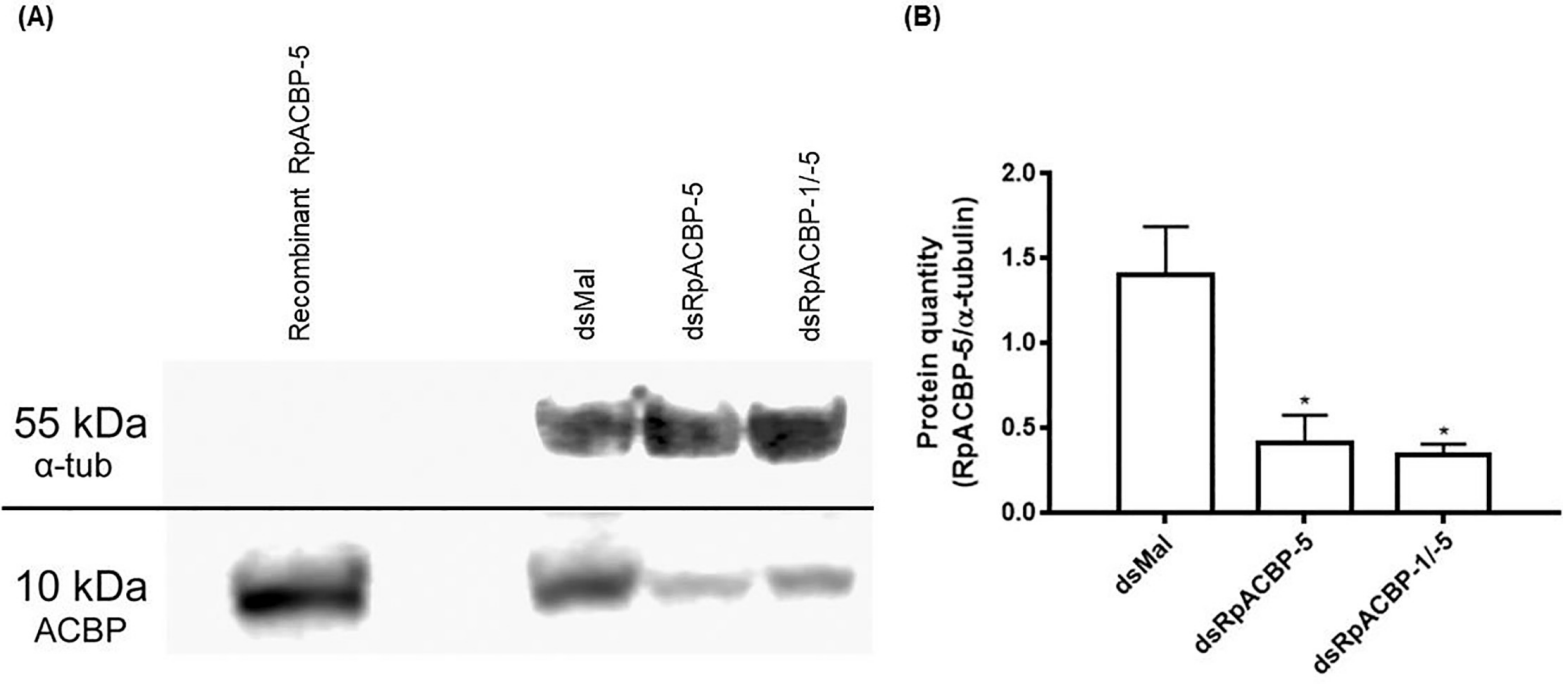
RpACBP-5 protein content in the posterior midgut after knockdown by RNAi. Fasted adult females were injected with 1 μg of dsRpACBP-5, or with a mixture of dsRNAs containing 1 μg of dsRpACBP-1 and 1 μg of dsRpACBP-5, or with two μg of dsMal, used as control. Insects were fed three days after injection, and the posterior midgut was collected on the seventh day after feeding. (A) Samples (60 μg protein) were subjected to Western blotting, with anti-RpACBP-5 antiserum or anti-α-tubulin antibody. The image is representative of three experiments. (B) The intensity of the bands was estimated by densitometry. The bars are means ± SEM. (*): significantly different from dsMal by one-way ANOVA, followed by Dunnett’s post-test with *p* < 0.05; n = 2–3.

After these results, the expression of both 10 kDa ACBPs (*RpACBP-1* and *RpACBP-5*) was simultaneously knocked down, in order to try to minimize a possible functional compensatory effect due to RpACBP-1 activity. As shown in Fig 7, the double knockdown significantly decreased the expression of both *RpACBP-1* and *RpACBP-5* in the posterior midgut, fat body, and ovary, up to 15 days after feeding. Additionaly, seven days after blood meal, the posterior midguts were analyzed by Western blot, in order to answer whether, by altering the mRNA abundance, the amount of protein would also be affected. As shown in Fig 8, the RpACBP-5 protein content was decreased to about one third, compared to control, when the insects were treated with either dsRpACBP-5 or dsRpACBP-1/5.

**Fig 9:**
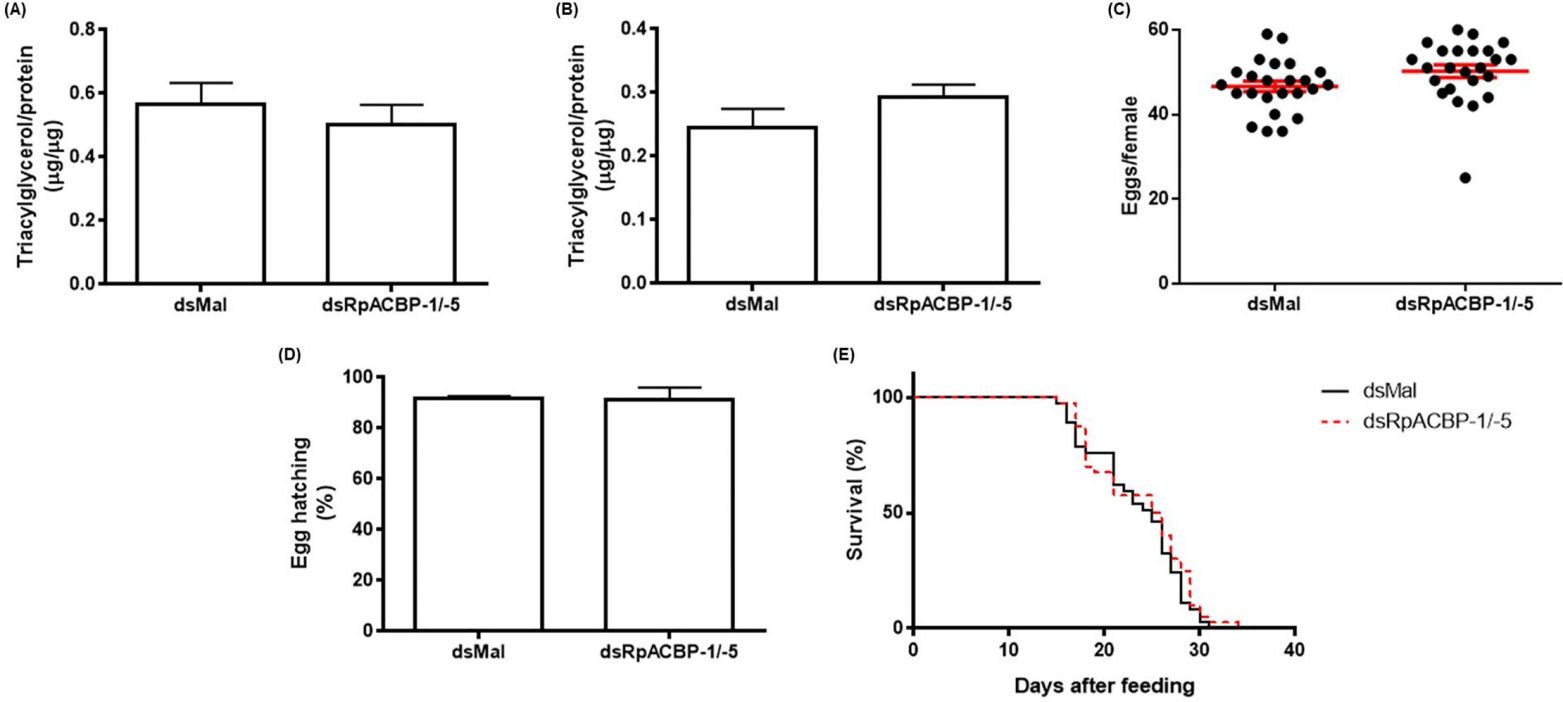
*RpACBP-1*/*RpACBP-5* double knockdown does not affect lipid accumulation, reproduction or lifespan. Adult fasted females were injected with a mixture of dsRNAs containing 1 μg of dsACBP-1 plus 1 μg of dsACBP-5 (dsRpACBP-1/5), or with 2 μg of dsMal, used as a control. The insects were fed three days after the injection. The symbols and bars indicate means ± SEM. The fat body (A) and oocytes (B) were collected on the seventh day after feeding, and the TAG content was determined. *p* > 0.05 by Student's *t*-test, n = 11–14. (C) Oviposition was monitored throughout the days after feeding. *p* > 0.05 by Student's *t*-test, n = 24. (D) Eggs were collected on the different days of the oviposition cycle. The total number of hatched eggs in each experimental group was determined. *p* = 0.9358 by χ^2^ test, n = 1205. (E) After feeding, insects were monitored daily. *p* > 0.05 by Log-Rank test, n = 24.

After double knockdown, the TAG content in the fat body and oocytes was determined seven days after blood meal, and no difference was observed when compared to control insects (Fig 9A and 9B). Reproduction was also evaluated, and neither the number of laid eeggs, nor the nymph hatching efficiency (around 90%), were affected by *RpACBP-1/5* silencing (Fig 9C and 9D). Subsequently, the insects were fasted to death, and survival percentage was recorded daily. Double knockdown again did not cause any effect on insect longevity (Fig 9E).

**Fig 10:**
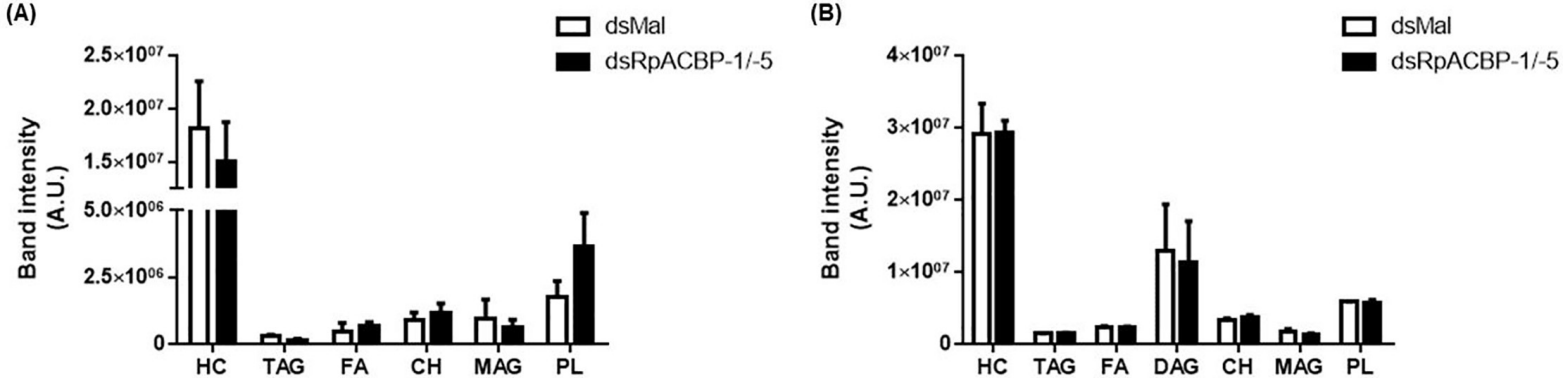
*RpACBP-1*/*RpACBP-5* double knockdown does not affect posterior midgut or hemolymph lipid composition. Starved females were injected with a mixture of dsRNAs containing one μg of dsACBP-1 plus one μg of dsACBP-5 (dsRpACBP-1/5), or with two μg of dsMal, used as a control. The insects were fed three days after the injection, and the posterior midgut (A) and hemolymph (B) were collected on the fourth day after feeding. Composition of total lipids was analyzed by HPTLC. *p* > 0.05 by Student's *t*-test, n = 3. HC = hydrocarbons; TAG = triacylglycerol; FA = fatty acid; DAG = diacylglycerol; CH = cholesterol; MAG = monoacylglycerol; PL = phospholipids.

**Fig 11:**
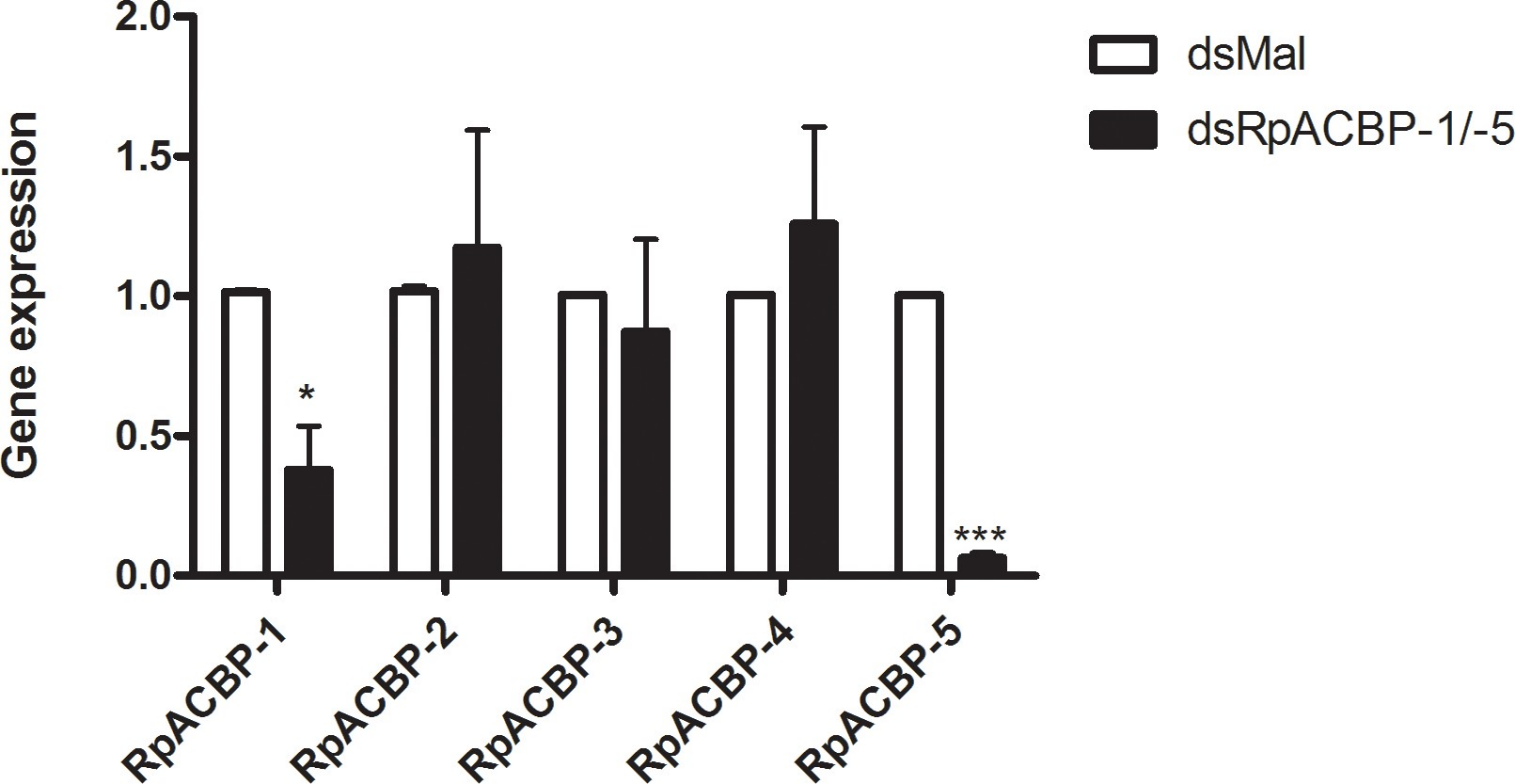
*RpACBP-1*/*RpACBP-5* double knockdown does not affect expression of the other *RpACBP* genes. Fasting adult females were injected with a mixture of dsRNAs containing 1 μg of dsACBP-1 plus 1 μg of dsACBP-5 (dsRpACBP-1/5), or with 2 μg of dsMal, used as a control. The insects were fed three days later and dissected at the fourth day after blood meal. Posterior midguts were obtained and homogenized. Samples were subjected to qPCR, for determination of *RpACBP-1*, *RpACBP-2*, *RpACBP-3*, *RpACBP-4*, and *RpACBP-5* mRNA levels, using *Rp18S* expression as reference gene. The bars are means ± SEM. (*), and (***): significantly different from dsMal by Student's *t*-test with *p* < 0.05 and 0.001, respectively; n = 4 (except: *RpACBP-3*, n = 3).

We then determined whether the lipid compositions of the hemolymph and posterior midgut were modified by the double knockdown. No significant differences were found between the two groups neither in the posterior midgut, nor in the hemolymph (Fig 10). As it was possible that some compensation in the expression of the other *RpACBP* genes had occurred after the *RpACBP-1/5* silencing, we determined their expression in the posterior midgut after the double knockdown, but no difference was observed (Fig 11).

## Discussion

Feeding induced *RpACBP-5* gene expression in the posterior midgut (Fig 2A) in a pattern that resembles the expression response described for the *RpACBP-1* gene [18]. Tenfold higher *RpACBP-1* mRNA levels were observed in the first day post-feeding, followed by a progressive decrease until the fourth day, when the level of expression was similar to that found in fasting insects [19]. However, these expression profiles had different time courses, as the *RpACBP-5* gene showed a slightly more delayed response to the effects of feeding. The ACBP expression in the midgut has already been described in some other insects, such as *Manduca sexta*, *D*. *melanogaster*, *Helicoverpa armigera* and *B*. *mori* [29–32]. Similarly, the ACBP expression in the midgut is also higher during the feeding periods of *M*. *sexta* and *H*. *armigera* caterpillars [31,32], suggesting this protein is important for lipid metabolism in intestinal epithelium.

The RpACBP-5 protein amount did not change throughout digestion in the posterior midgut, being detected in high amounts in all analyzed days (Fig 1B and 1C), despite the increase in mRNA after feeding. It is unknown why there is no correlation between the mRNA and protein levels, however this result raised two possibilities. First, the RpACBP-5 half-life can be long, making it impossible to detect oscillations in proteins levels. Another hypothesis is that the protein degradation rate increases after the blood meal, and the observed transcriptional increase keeps the protein levels constant. This same mismatch between protein and mRNA levels was also observed in the fifth instar larvae of *M*. *sexta*, where the ACBP gene expression peaked between days two and three after molting, abruptly declining to levels almost impossible to detect in later days, while the protein reached its maximal levels on the fourth day after the molt [31].

In the ovarian follicles, the *RpACBP-5* gene transcript levels were highest in the initial part of oogenesis, and then were gradually reduced as oocyte growth continued (Fig 2B). This result was similar to the expression profile of some other genes related to lipid metabolism in this insect, such as acyl-CoA synthetase 2 [33] and diacylglycerol acyltransferase [34]. *RpACBP-5* gene expression in the ovary was previously shown to be similar, not significantly different, from the midgut [18]. In this way, it is noteworthy that RpACBP-5 protein was detected only in the insect posterior midgut, and surprisingly not in the ovary (Fig 1A). Considering that the ovary has exceptional high amounts of vitellin [35], probably the visualization of ACBP in the same gel and conditions of other samples was not possible, due to tiny protein quantity, in comparison to the main ovary protein.

The binding of acyl-CoA to recombinant RpACBP-5 was studied by native gel migration assay and calorimetry (Fig 3A). RpACBP-5 was able to bind all tested acyl-CoA, ranging in length from 10 to 26 carbons, with up to 3 unsaturations and odd number of carbons, as previously also shown for RpACBP-1 [18] and for ACBPs from other organisms [36–38]. Calorimetry analysis showed that RpACBP-5 interacts with only one ligand molecule, exhibiting stoichiometry of 1:1 mol/mol (Fig 3B). The same was described in other models [39–41]. The change in enthalpy of -8.1 kJ/mol shows that the binding is exothermic, which is similar to previously published values for bovine recombinant ACBP titrated with dodecanoyl-CoA [26]. The obtained *K*_*d*_ value was 4.0 × 10^−7^ M. Some considerable variations are known to occur in *K*_*d*_ values for interaction of ACBPs and acyl-CoAs, as it is the case, for instance, for the bovine ACBP. The *K*_*d*_ value of the binding between bovine ACBP and hexadecanoyl-CoA measured by Rasmussen et al. (1994) was 0.45 x 10^−13^ M, showing that ACBP has higher affinity for longer acyl-CoAs. When measured with dodecanoyl-CoA, the bovine ACBP affinity was determined to be 1.7 x 10^−8^ M, similar to the one described here for RpACBP-5. Therefore, it is worth emphasizing that differences regarding ACBP affinity for acyl-CoA are expected when either different ACBPs or ligand lipids of different sizes are compared. However, even with variations in *K*_*d*_ values, they still remain in the nanomolar range, which shows that ACBP binds different acyl-CoAs with very high affinity. These results show that, like RpACBP-1 [18], RpACBP-5 encodes a functional ACBP.

The effects caused by RpACBP-5 knockdown by RNAi were analyzed, in order to continue the characterization of the functions that the RpACBP-5 protein may have in lipid metabolism in the kissing-bug. As *RpACBP-5* has a high expression in the midgut, this protein could play an essential role in the absorption of FA that are generated by the activity of luminal lipases after feeding [42], what could affect blood digestion, in a general way. So, the protein content of total midgut was determined, as an overall indicative of the digestive process (Fig 6A). No difference was observed, indicating that *RpACBP-5* knockdown did not impact blood digestion. A similar result was observed with *RpACBP-1* knockdown, when females showed no significant difference in body mass decrease, used in that case to follow digestion [18]. These results indicate that the overall digestive process was not affected, but more accurate experiments would be necessary for the specific evaluation of lipid absorption so that more detailed conclusions regarding this point could be reached.

The TAG levels in the fat body of knockdown insects were investigated on the seventh day after feeding (Fig 6B), considering that TAG represents the main component of lipid droplets in eukaryotes [43]. We had previously shown that the TAG content stored in the fat body remains high and constant between the fourth and 13^th^ day after feeding [44]. The *RpACBP-5* knockdown did not affect the amount of TAG in the fat body, similarly to the result obtained after *RpACBP-1* knockdown [18].

The ovary also had high *RpACBP-5* expression [18]. Oogenesis begins a few hours after the blood meal and, from that time, the ovary already demands a large amount of lipids for egg production [45]. As oocytes grow, they accumulate lipids mainly as TAG [46], that may originate from the diet or from fat body depots, depending on the metabolic condition. So, we considered that oogenesis could be affected by *RpACBP-5* knockdown. Both TAG content in oocytes, and the number of laid eggs remained the same (Fig 6C and 6D), showing that the knockdown did not interfere with the lipid availability required for egg production. In the same way, *RpACBP-5* knockdown did not affect egg viability, as hatching proportion was the same (Fig 6E). Besides, female survival during prolonged starvation was not altered (Fig 6F). These data indicate that RpACBP-5 is not an essential protein for maintaining life. Similarly, *RpACBP-1* knockdown did not affect the viability of the laid eggs, neither the survival curve of the knockdown females, corroborating the data described above [18]. A similar result was reported in *B*. *mori*, in which ACBP knockdown in either the pheromone gland or the midgut did not affect pupal development or adult insect emergence [14]. Although it was not observed any difference in gene expression of the other RpACBP proteins that could explain the absence of phenotypes after inhibition of *RpACBP-5* expression (Fig 5), it is possible that the other components of ACBP family show a functional compensatory response. In this way, the insect would overcome the challenges presented by the *RpACBP-5* knockdown, as one or more of the other RpACBP proteins might accomplish its roles.

Since a single ACBP knockdown did not cause a relevant disturbance in insect physiology, we considered that the combined knockdown of more isoforms could cause some phenotype not previously observed. In that way, a double knockdown of *RpACBP-1* and *RpACBP-5* was performed. The content of TAG in the fat body and chorionated oocytes, as well as oviposition, egg hatching rate, and longevity of the insects were not altered (Fig 9). It was expected that the decrease in levels of ACBPs would disrupt the dietary FA flow, and that this change would have effects on insect lipid metabolism, affecting the accumulation of TAG in the fat body. However, no changes were noticed.

More detailed lipid analyses were carried out due to the absence of phenotypes. Surprisingly, the double knockdown did not affect the lipid profile in any of the analyzed samples (Fig 10). Considering that gene expression of the other RpACBP proteins in the posterior midgut was not affected by the *RpACBP-5/1* knockdown (Fig 11), we hypothesize that no phenotypes were detected because any of the other three isoforms might play a role similar to the knockdown proteins. For instance, *RpACBP-2*, *RpACBP-3*, *RpACBP-4*, and *RpACBP-5* genes have transcript levels that do not differ significantly from each other but are higher than the *RpACBP-1* gene in the ovary [18], suggesting that any of these could be acting in oogenesis maintenance. The similar expression profile reinforces the idea that the same function may be performed by another isoform, according to the situation, preventing the identification of effects in the insect physiology.

Previous data pointed that the *RpACBP-1* knockdown increases TAG and decreases DAG levels in the posterior midgut [18], but the same result was not obtained after *RpACBP-1* and *-5* double knockdown. However, the comparison between these results is difficult, since the samples from the study mentioned above were processed along with the intestinal contents, making it impossible to confirm that the lipid levels obtained in the mass spectrometry came from the intracellular stores. Also, other classes of neutral lipids were not analyzed in that study, what affects the composition profile of the main classes of identified lipids.

We have also attempted to knockdown all five ACBPs, using a mixture of dsRNA. However, the resulting knockdown was very variable and not reproducible, which did not allow us to proceed with the analysis of possible phenotypes.

In summary, this study shows that the blood feeding induces *RpACBP-5* gene activity in the posterior midgut, but protein levels remain stable. Also, this gene is highly expressed in the pre-vitellogenic follicles and early vitellogenic oocytes. *In vitro* analysis showed that RpACBP-5 is a functional ACBP, capable of binding acyl-CoA with high affinity. However, our reverse genetics experiments did not indicate the roles of this protein *in vivo*. These results lead us to believe that the lack of differences between control and knockdown insects may be an effect of an overlapping of functions with the other proteins of the ACBP family present in *R*. *prolixus*.

## Supporting information

S1 Fig Assay to test antiserum specificity against RpACBP-5. Amounts ranging from 10 μg to 1 ng of the recombinant RpACBP-5 protein were applied onto a nitrocellulose membrane, which was incubated with anti-RpACBP-5 antiserum and developed with ECL. Recombinant RpACBP-1 (10 μg) was used to check the antiserum specificity.
(TIF)

S2 Fig Original images that were used for building figures that present blots (Fig 1A and 1B, Fig 8A, and in S1 Fig). (PDF)

S1 Table Primer sequences used for qPCR amplification. List of all primer sequences that were used for gene expression determinations by qPCR.
(DOCX)

S2 Table Additional information on the primers. Detailed information on properties of primers that were used in qPCR experiments.
(DOCX)

S3 Table Primer sequences used for dsRNA synthesis. List of primer sequences that were used for the synthesis of dsRNA, used in knockdown experiments.
(DOCX)
