# Supplementary figures and images for: Expression of acyl-CoA-binding protein 5 from Rhodnius prolixus and its inhibition by RNA interference
Source: PLoS One. 2020 Jan 14;15(1):e0227685. doi: 10.1371/journal.pone.0227685 (PMC6959561; doi:10.1371/journal.pone.0227685)

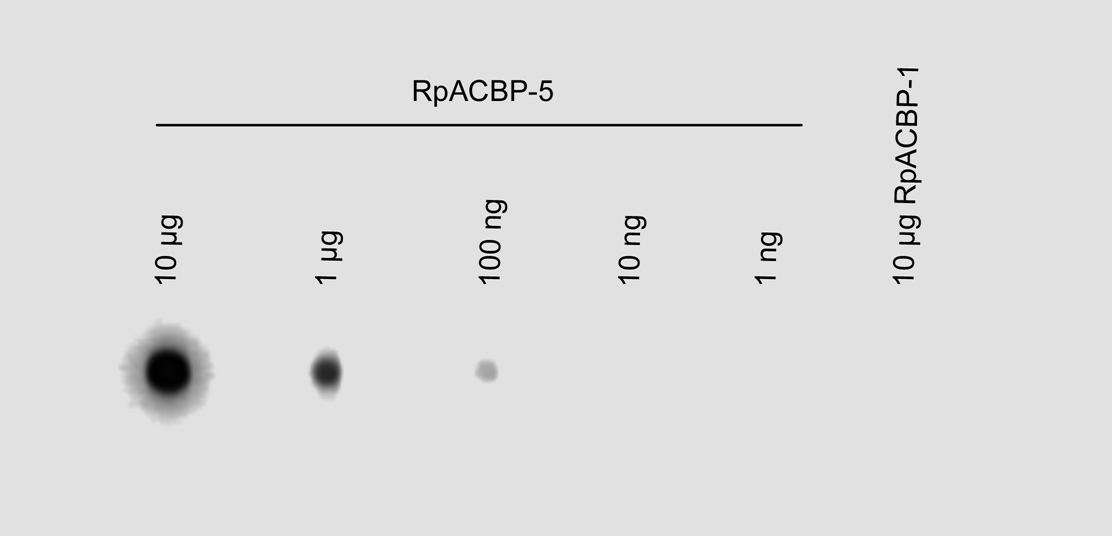

Supplement: S1 Fig — Amounts ranging from 10 μg to 1 ng of the recombinant RpACBP-5 protein were applied onto a nitrocellulose membrane, which was incubated with anti-RpACBP-5 antiserum and developed with ECL. Recombinant RpACBP-1 (10 μg) was used to check the antiserum specificity. (TIF) [file pone.0227685.s001.tif]
