# Supplementary material for: Expression of acyl-CoA-binding protein 5 from Rhodnius prolixus and its inhibition by RNA interference
Source: PLoS One. 2020 Jan 14;15(1):e0227685. doi: 10.1371/journal.pone.0227685 (PMC6959561; doi:10.1371/journal.pone.0227685)
Supplement: S2 Fig — (PDF) [file pone.0227685.s002.pdf]

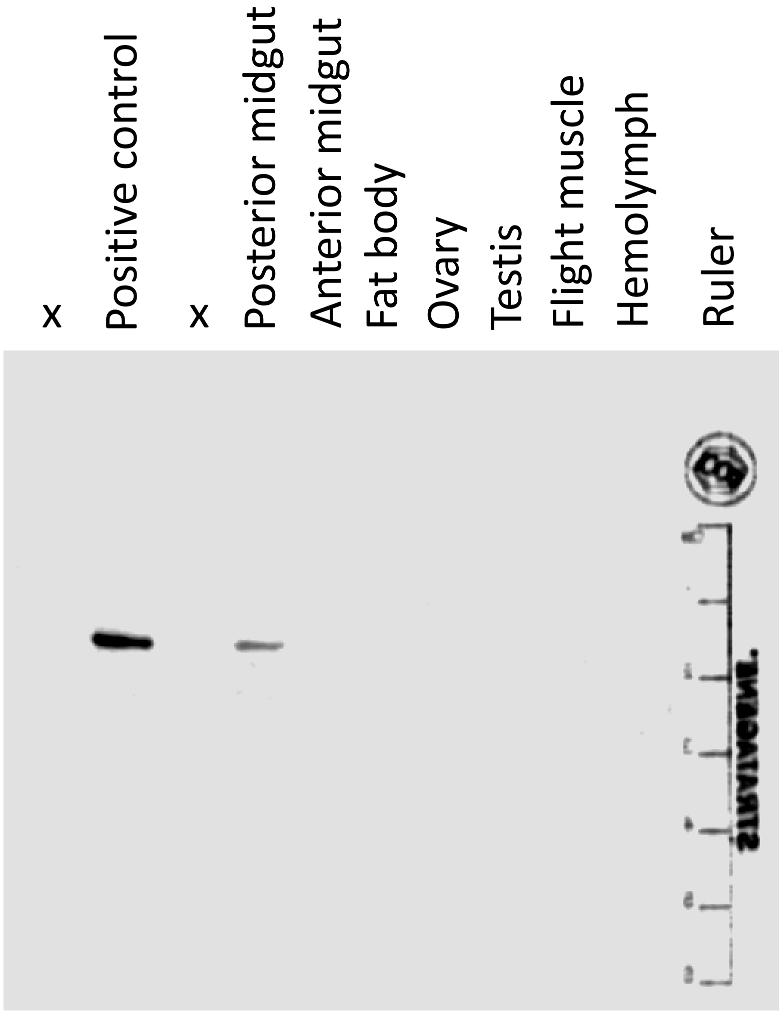

RpACBP-5; captured with LI-COR C-Digit scanner

Almeida et al., 2019; Figure 1A

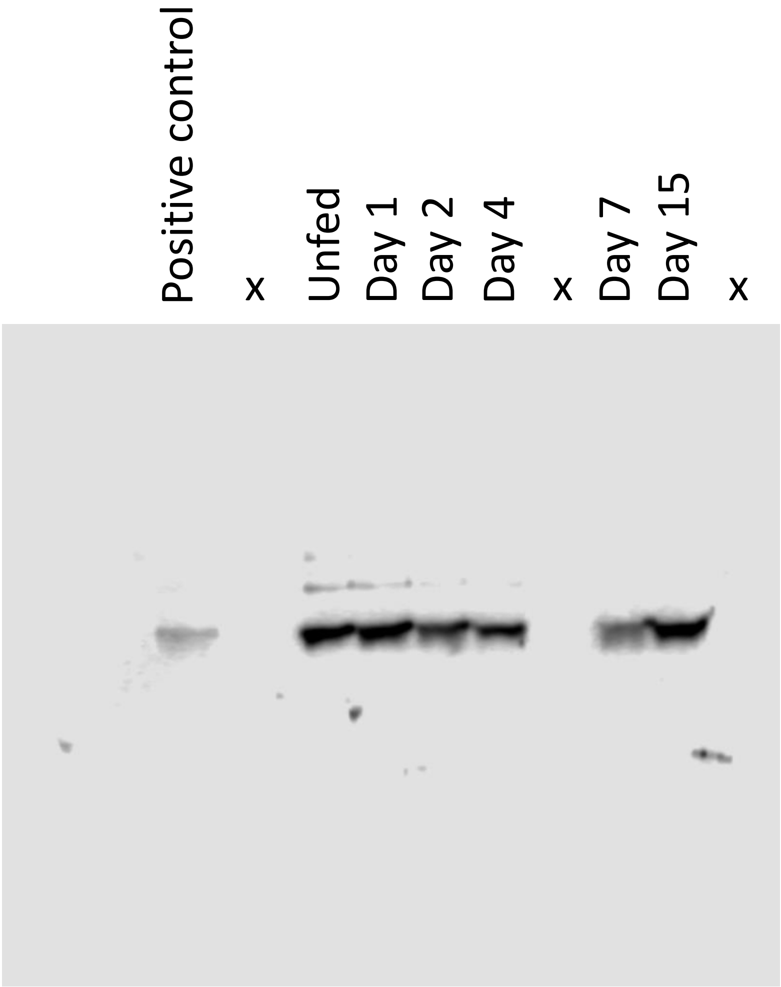

RpACBP-5; captured with LI-COR C-Digit scanner

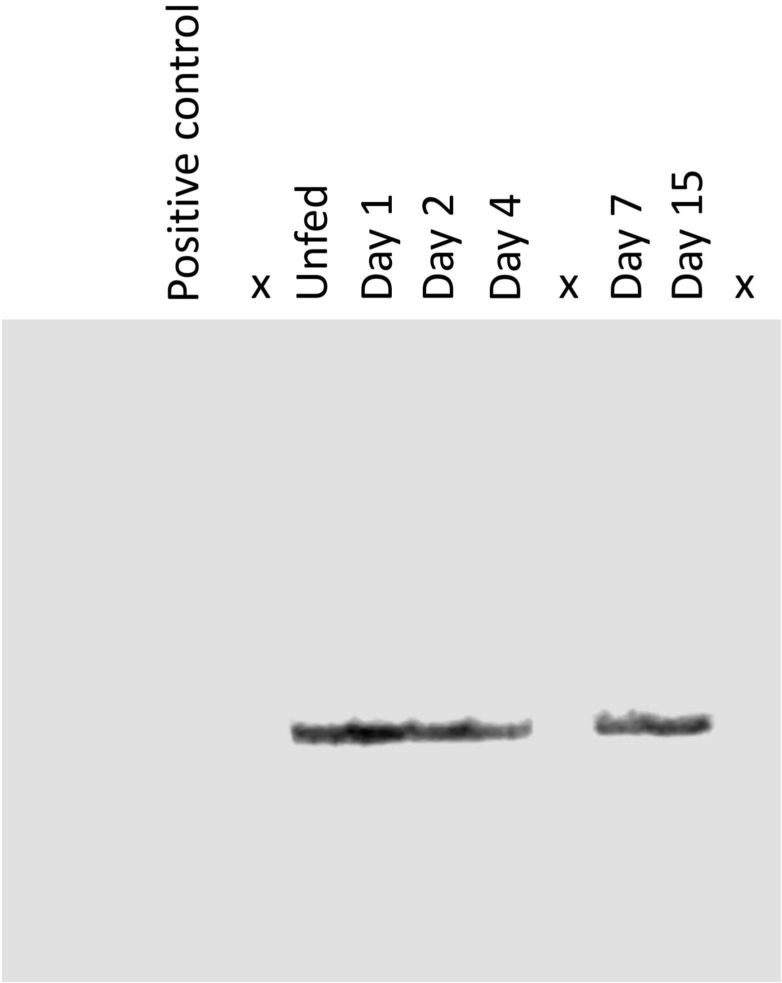

α-tubulin; captured with LI-COR C-Digit scanner

Almeida et al., 2019; Figure 1B

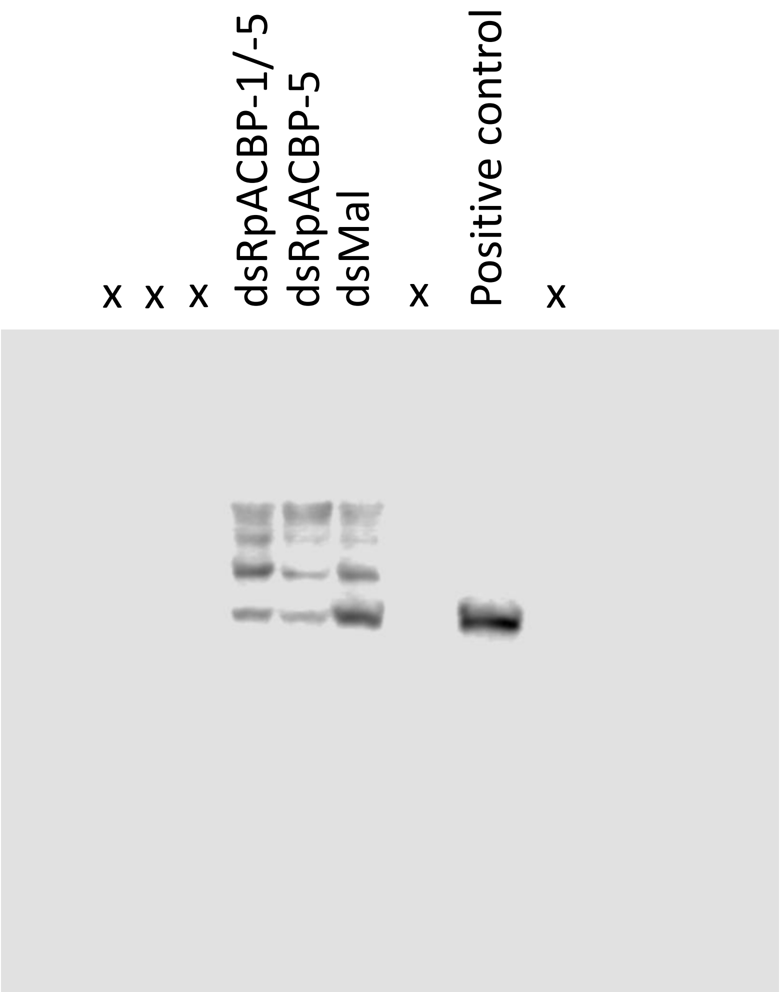

RpACBP-5; captured with LI-COR C-Digit scanner

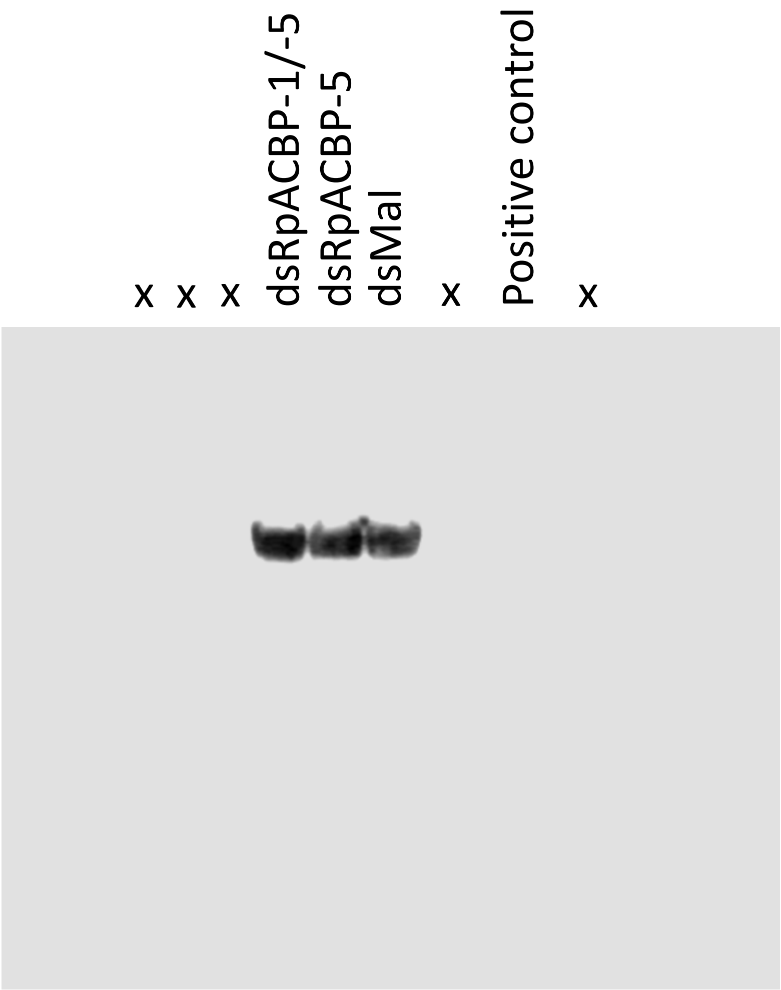

α-tubulin; captured with LI-COR C-Digit scanner

Almeida et al., 2019; Figure 8A

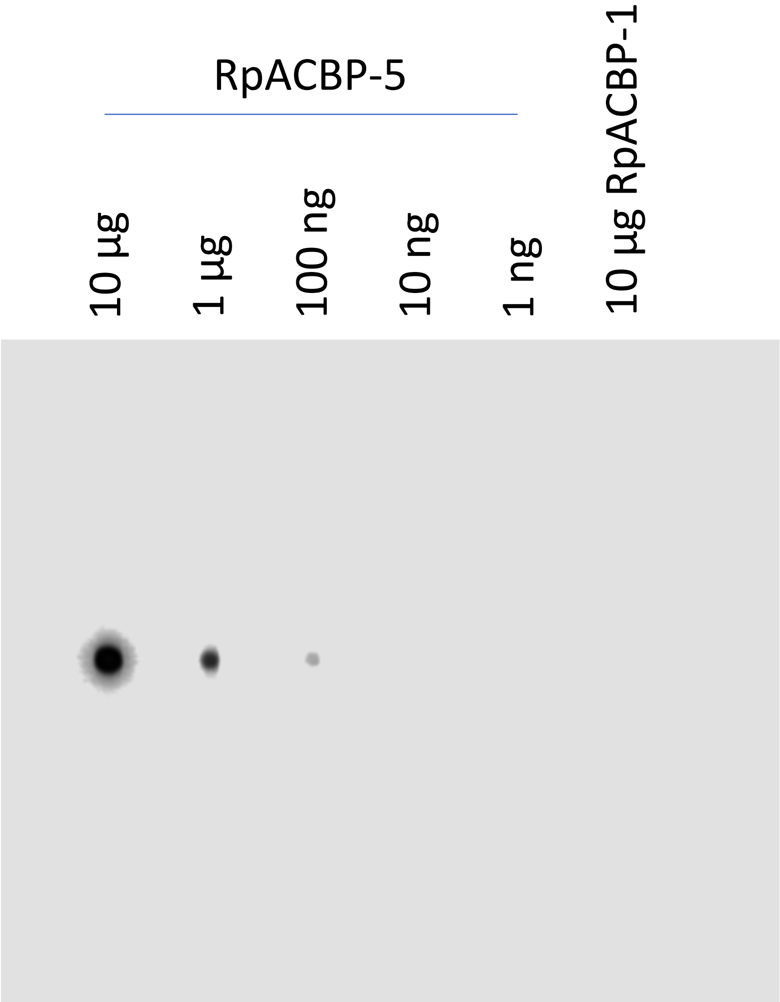

RpACBP-5; captured with LI-COR C-Digit scanner

Almeida et al., 2019; Figure S1
