# Supplementary material for: Expression of acyl-CoA-binding protein 5 from Rhodnius prolixus and its inhibition by RNA interference
Source: PLoS One. 2020 Jan 14;15(1):e0227685. doi: 10.1371/journal.pone.0227685 (PMC6959561; doi:10.1371/journal.pone.0227685)
Supplement: S1 Table — List of all primer sequences that were used for gene expression determinations by qPCR. (DOCX) [file pone.0227685.s003.docx]

**S1_Table. Primer sequences used for qPCR amplification.**

| **Primer** | **Sequence (5' - 3')** |
| --- | --- |
| *RpACBP-1_F* | GGGGACTGTAATACGAGCAA |
| *RpACBP-1_R* | TTCAATCCATAAGATGCAATCA |
| *RpACBP-2_F* | TACAGGACAAACTGCGTTGC |
| *RpACBP-2_R* | ATCATTGCAAGCCACATTCA |
| *RpACBP-3_F* | AAGCCACAGAGGGAGTAGCA |
| *RpACBP-3_R* | TTACCTAATTTGGCCCATGC |
| *RpACBP-4_F* | GTCCGAGAGTGAGGACGAAG |
| *RpACBP-4_R* | CTACTCCAGCCATCCTCGAC |
| *RpACBP-5_F* | CAACACAGCAAAACCTGGAG |
| *RpACBP-5_R* | CCATCGCTGATTCTTTGCTT |
| *Rp18S_F* | TGTCGGTGTAACTGGCATGT |
| *Rp18S_R* | TCGGCCAACAAAAGTACACA |
| *RpEF-1_F*  *RpEF-1_R* | GATTCCACTGAACCGCCTTA  GCCGGGTTATATCCGATTTT |


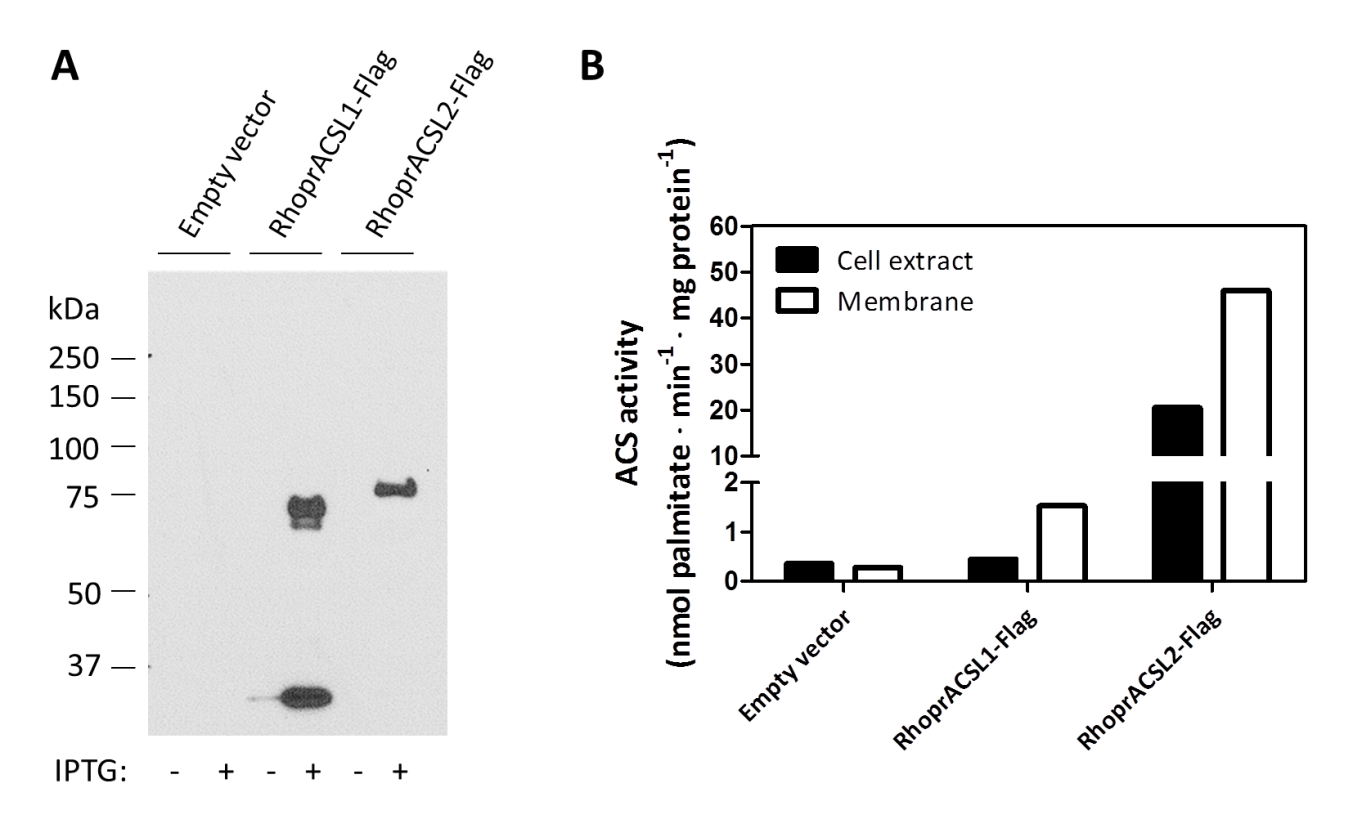


**Supplementary** **Figure 1: Protein expression and ACS activity of recombinant RpACSL1- and RpACSL2-FLAG.**

*RpAcsl1* and *RpAcsl2* full-length CDSs were cloned into pFLAG-CTS expression vector and used for *E. coli* (JM109 strain) transfection. (A) Whole cell extracts were obtained from bacteria transfected with empty vector, RpACSL1- or RpACSL2-FLAG plasmids before (0h) or 4 h after IPTG induction. Samples were subjected to 8% SDS-PAGE followed by Western blot assay using anti-FLAG M2 antibody. (B) Whole cell extracts (2-6 µg) or membrane enriched fractions (1-3 µg) obtained from transfected bacteria were assayed for ACS activity.


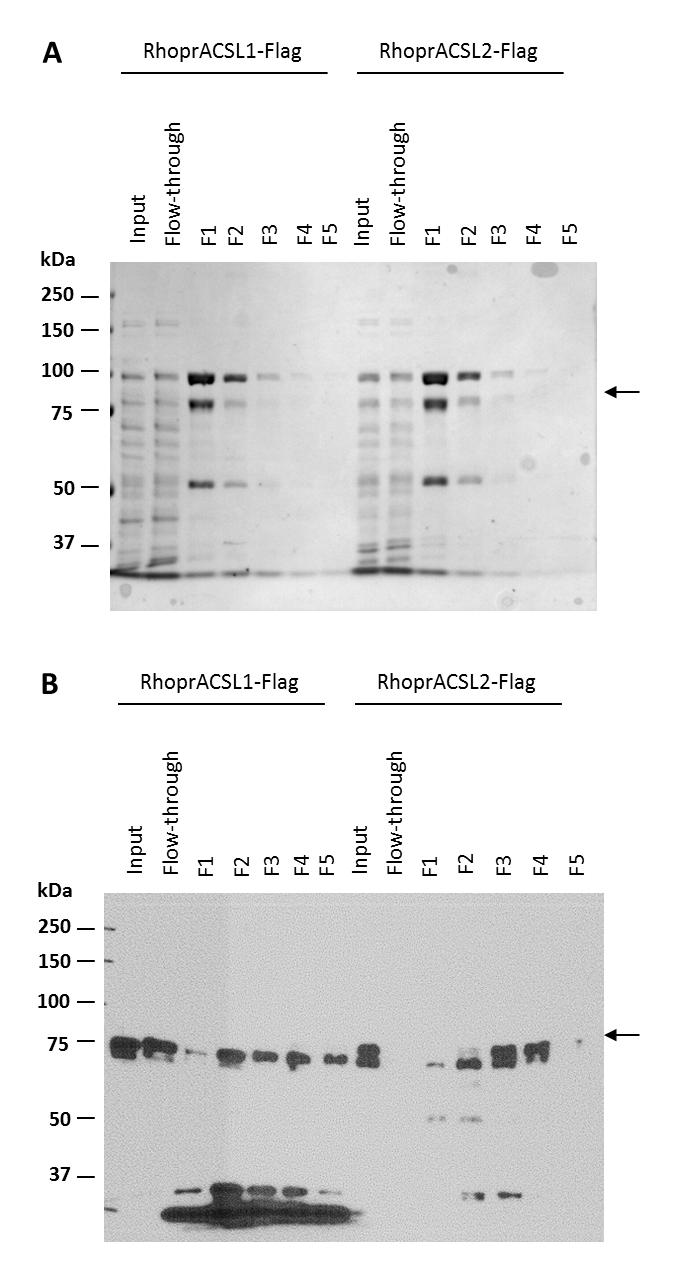


**Supplementary** **Figure 2: Purification of recombinant RpACSL1- and RpACSL2-FLAG.**

*E. coli* (JM109 strain) was transfected with pFLAG-CTS containing RhoprAcsl1- or RhoprAcsl2-Flag inserts.. Recombinant proteins were purified from membrane enriched fractions by column chromatography. Eluted fractions (F 1-5) were subjected to 8% SDS-PAGE (A) followed by Western blot assay using anti-FLAG M2 antibody (B). Whole cell extracts (2-6 µg) or membrane enriched fractions (1-3 µg) obtained from transfected bacteria were assayed for ACS activity. Results are representative for 3 independent determinations. Arrow indicates molecular mass predicted for RhoprACSL1-Flag and RhoprACSL2-Flag recombinant proteins (81 and 83 kDa, respectively).


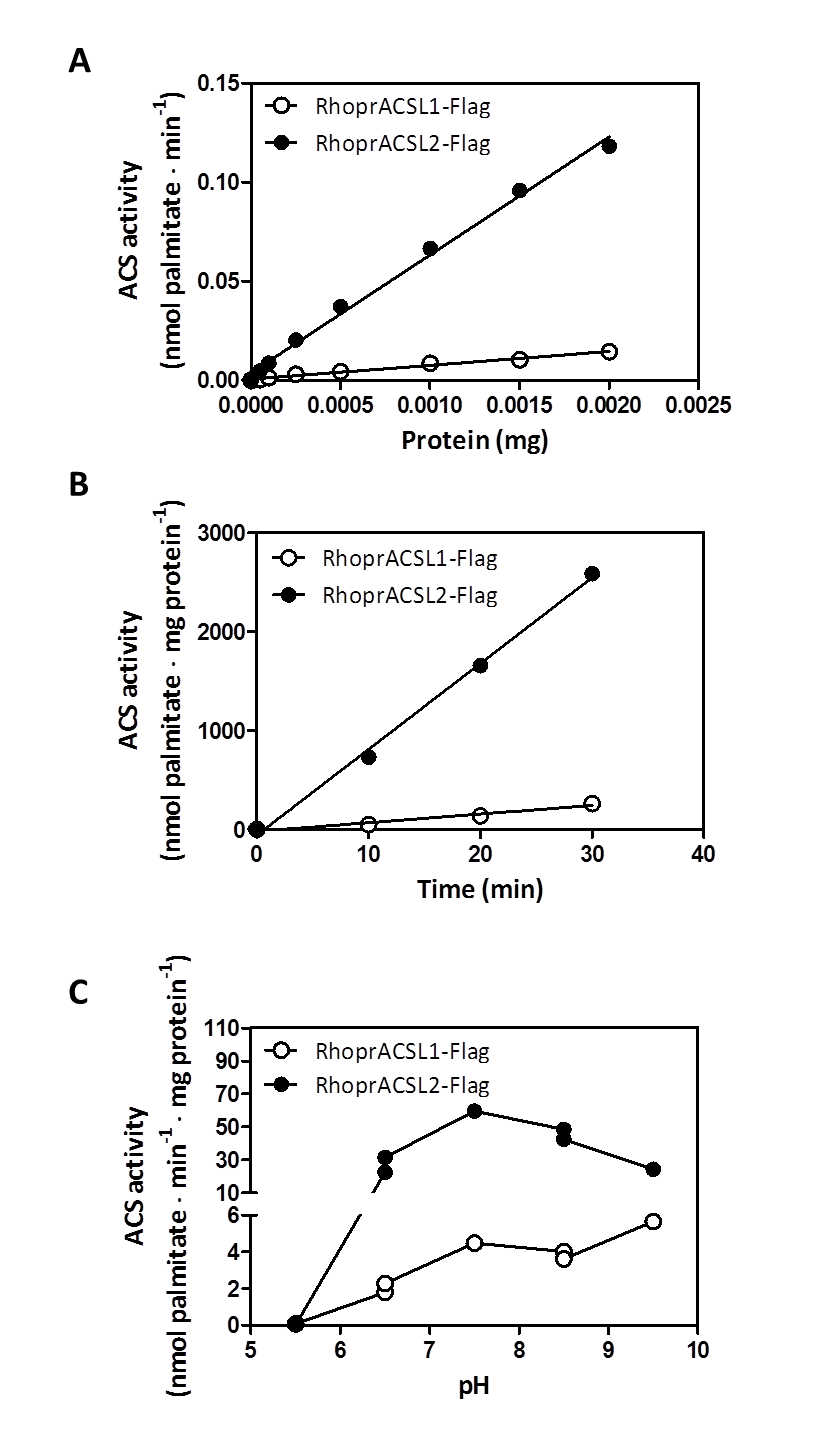


**Supplementary** **Figure 3: ACS dependence on protein amount, incubation time and pH.**

(A) Flag-purified RpACSL1- and RpACSL2-FLAG were assayed for ACS activity in the presence of varying concentrations of protein for 10 min at 25°C and pH 7.5. (B) Flag-purified RpACSL1- (0.25 - 1 µg) and RpACSL2-FLAG (0.1 – 0.5 µg) were assayed for ACS activity for 10-30 min at 25°C and pH 7.5. (C) Flag-purified RpACSL1- (0.25 - 1 µg) and RpACSL2-FLAG (0.1 – 0.5 µg) were assayed for ACS activity for 10 min at 25°C and the pH of the reaction media was varied as indicated. MES was used as a buffer for ACS activities from pH 5.5-6.5, Tris for pH 6.5–8.5, and glycine for pH 8.5 and 9.5. All the assays were performed with 50 µM palmitate. Results are representative for 3 independent determinations.


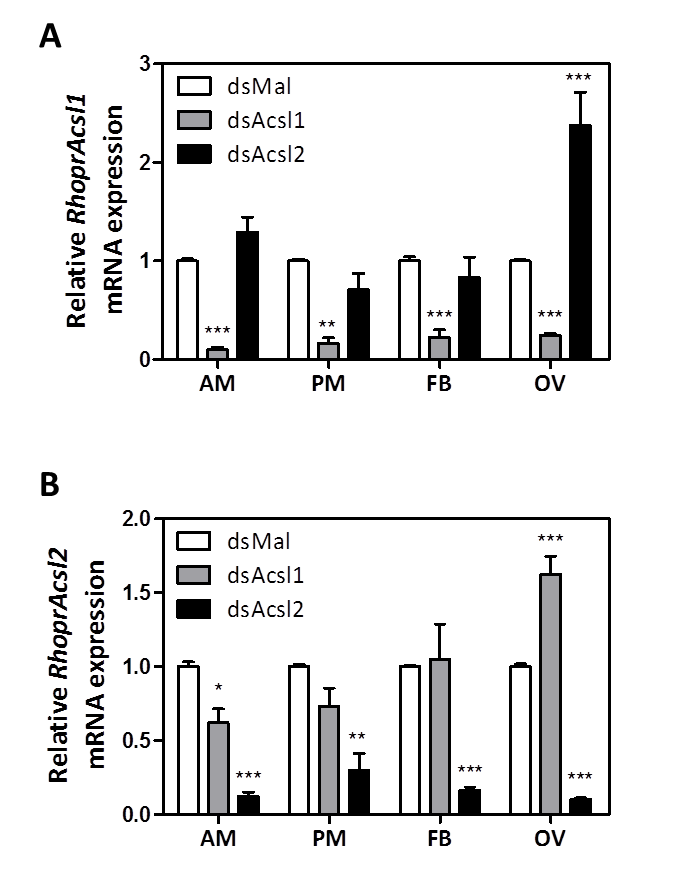


**Supplementary Figure 4: Inhibition of RhoprAcsl1 and RhoprAcsl2 in different organs of *R. prolixus*.**

Fasted adult females were injected with 1 µg of dsRNA for *RhoprAcsl1*, *RhoprAcsl2 o*r *Mal* (control) genes. Insects were fed on the third day after injection. Ten days after feeding, the total RNA was obtained from anterior and posterior midguts, fat body and ovaries. Differences between cDNA levels were determined by qPCR using specific primers designed for *RhoprAcsl1*or *RhoprAcsl2*. *Rhopr18S* was used as a reference gene. Gene expression levels are expressed relative to dsMal injected insects. Results are mean±S.E.M. for 3 independent determinations. (*), (**) and (***): P<0.05, P<0.01 and P<0.001, respectively, compared to control group by one-way ANOVA. AM, anterior midgut; PM, posterior midgut; FB, fat body; OV, ovary.
