# Supplementary material for: Expression of acyl-CoA-binding protein 5 from Rhodnius prolixus and its inhibition by RNA interference
Source: PLoS One. 2020 Jan 14;15(1):e0227685. doi: 10.1371/journal.pone.0227685 (PMC6959561; doi:10.1371/journal.pone.0227685)
Supplement: S2 Table — Detailed information on properties of primers that were used in qPCR experiments. (DOCX) [file pone.0227685.s004.docx]

**S2_Table. Additional information on the primers.**

| Gene | Amplicon size (bp) | Efficiency (%) | Curve slope | *y* intercept | *r*^2^ |
| --- | --- | --- | --- | --- | --- |
| *RpACBP-1* | 152 | 137.0 | -2.7 ± 0.2 | 31.0 ± 0.2 | 0.9783 |
| *RpACBP-2* | 103 | 91.9 | -3.5 ± 0.3 | 22.3 ± 0.6 | 0.9250 |
| *RpACBP-3* | 91 | 73.7 | -4.2 ± 0.3 | 23.1 ± 0.6 | 0.9395 |
| *RpACBP-4* | 101 | 65.8 | -4.6 ± 0.5 | 24.2 ± 0.9 | 0.8940 |
| *RpACBP-5* | 95 | 103.6 | -3.2 ± 0.0 | 22.3 ± 0.1 | 0.9992 |
| *Rp18S*  *RpEF-1* | 104  92 | 100.2  93.0 | -3.3 ± 0.1  -3.5 ± 0.1 | 4.94 ± 0.2  17.4 ± 0.1 | 0.9936  0.9979 |
