# Supplementary material for: Expression of acyl-CoA-binding protein 5 from Rhodnius prolixus and its inhibition by RNA interference
Source: PLoS One. 2020 Jan 14;15(1):e0227685. doi: 10.1371/journal.pone.0227685 (PMC6959561; doi:10.1371/journal.pone.0227685)
Supplement: S3 Table — List of primer sequences that were used for the synthesis of dsRNA, used in knockdown experiments. (DOCX) [file pone.0227685.s005.docx]

**S3_Table. Primer sequences used for dsRNA synthesis.**

|  |  | |
| --- | --- | --- |
| *RpACBP-1_F* | | TAATACGACTCACTATAGGGTACTATGACAGAAGAGGGT |
| *RpACBP-1_R* | | TAATACGACTCACTATAGGGTACTTTATAGTTTCAATCC |
| *RpACBP-5_F* | | TAATACGACTCACTATAGGTTACTATGGCGCTAGTAGAGAGATT |
| *RpACBP-5_R* | | TAATACGACTCACTATAGGGTACTTTTTCCCAAGCTTCCCATTT |
| *T7 minimal* | | TAATACGACTCACTATAGGG |
|  | | |
